# Phenotypic Characterization and Whole-Genome Analysis of a Novel Bacteriophage HCF1 Infecting *Citrobacter amalonaticus* and *C. freundii*

**DOI:** 10.3389/fmicb.2021.644013

**Published:** 2021-01-25

**Authors:** Prince Kumar, Mukesh K. Meghvansi, Dev V. Kamboj

**Affiliations:** Biotechnology Division, Defence Research and Development Establishment, Gwalior, India

**Keywords:** *Citrobacter amalonaticus*, bacteriophage, *C. freundii*, whole-genome analysis, phenotypic characterization

## Abstract

*Citrobacter* species often occur in sewage, food, soil, wastewater, and in the intestinal tract of animals and humans. *Citrobacter* spp. cause urinary tract infections (UTIs) and infantile meningitis in humans. Due to the presence of plasmid-encoded resistance genes, *Citrobacter* spp. are often resistant to many antibiotics. In this study, *Citrobacter* virus HCF1, a novel virulent bacteriophage capable of killing *Citrobacter amalonaticus* and *Citrobacter freundii*, was isolated from the sewage water. The isolated bacteriophage was characterized with respect to transmission electron microscopy, one-step growth curve, host range, *in vitro* efficacy, storage stability, and environmental stress tolerance. The one-step growth curve analysis revealed that the latent period of HCF1 was 30 min and the estimated burst size was 121 plaque-forming units (PFU) per bacterial cell. Host range testing indicated that the HCF1 was specific to the *Citrobacter* genus. *In vitro* efficacy assay in the effluent of an anaerobic biodigester showed that the HCF1 completely eliminated the host within 4 and 5 h at MOI:100 and MOI:10, respectively, thereby indicating its potential for combating *C. amalonaticus* infections. The isolated bacteriophage is considerably stable and tolerant to environmental stress. Furthermore, the complete genome of HCF1 was sequenced using Oxford Nanopore sequencing and the data were subjected to detailed bioinformatic analyses. NCBI-BLASTn analysis revealed that the HCF1 genome had a query coverage of 15–21% and a maximum similarity of 77.27–78.49% with 11 bacteriophages of the *Drexlerviridae* family. Detailed bioinformatic analysis of the genome profile suggests that HCF1 is a novel *T1svirus* belonging to the *Tempevirinae* subfamily of the *Drexlerviridae* family.

## Introduction

*Citrobacter* is a genus of aerobic, Gram-negative bacteria belonging to the *Enterobacteriaceae* family. *Citrobacter* spp. have been reported from sewage (*Citrobacter rodentium*, Mizuno et al., 2020; *Citrobacter freundii*, Kim et al., 2020), water bodies (*Citrobacter werkmanii* and *Citrobacter amalonaticus*, Royam and Nachimuthu, 2020), human gut (*C. freundii*, Nada et al., 2004), and animal intestines (*C. werkmanii* and *C. freundii*, Bae et al., 2010). The presence of *Citrobacter* spp. in the wastewater poses a serious public health threat, especially in large human populations, because these bacteria can spread very easily and quickly once infection occurs. Infection with *Citrobacter* spp. is primarily associated with gastroenteritis, neonatal meningitis, and urinary tract infections (UTIs) (Lipsky et al., 1980). In a retrospective analysis carried out from January 2009 to December 2010 using urine samples from 4126 patients in India, *Citrobacter* was found to be the third most common causative agent of UTIs in hospitalized patients, next only to *Escherichia coli* and *Klebsiella* spp., accounting for up to 9.4% of all isolates (Peerapur et al., 2013). Similarly, in another study (Sami et al., 2017) conducted in Northern India, among 246 patients who tested positive for *Citrobacter* spp., *C. amalonaticus* was reported in 9.75% of the patients. A study (Garcia et al., 2016) reporting four cases of *C. amalonaticus* infection in patients hospitalized in Marseille, France, using MALDI-TOF technique observed statistically higher prevalence of the UTI episodes, thereby indicating that this bacterium could be an emerging pathogen responsible for UTIs in immunocompromised patients. These studies suggest that *C. amalonaticus* could be a potential pathogen and a causative agent of UTIs in immunocompromised patients worldwide.

Study (Garcia et al., 2016) of *C. amalonaticus* infection reported in Marseille, France, suggested that the bacterial isolates associated with various cases exhibited resistance to different antibiotics such as amoxicillin, ticarcillin, cotrimoxazole, amoxicillin/clavulanate, and ticarcillin/clavulanate. Although *Citrobacter* spp. are regarded as rare human pathogens, this genus contains plasmid-encoded resistance genes which confer considerable resistance to many antibiotics. Therefore, *Citrobacter* spp. pose a significant public health threat to hospital patients as well as the general public. In a study of 36 patients with *C. freundii* bacteremia in a tertiary medical center in northern Taiwan between 2009 and 2014, a high degree of antibiotic resistance was reported for various first- and second-generation antibiotics including cefuroxime (66.7%), cefoxitin (97.2%), and cefazolin (100%). Furthermore, polymerase chain reaction (PCR)-based detection of resistance genes in these bacterial isolates suggested the presence of β-lactamase genes (Liu et al., 2017). More recently, carbapenemase-producing *C. amalonaticus* strain has been reported from hospital sewage and river water site in the Philippines (Suzuki et al., 2020). Multidrug resistant (MDR) bacteria of public health importance assume greater significance in the wake of reports from various parts of the world indicating that *Citrobacter* spp. can produce New Delhi metallo-β-lactamase 1 (NDM-1), a mediator of carbapenem resistance. A recent study from a 22-bed digestive rehabilitation center in Buenos Aires (Argentina) reporting an episode of a sudden 2-week outbreak of *C. amalonaticus* infection revealed the presence of *bla*NDM-1 strain (Royer et al., 2019). Similarly, another study (Faccone et al., 2019) reported an MDR isolate, identified as *C. amalonaticus* using MALDI-TOF MS and confirmed by genomic analysis, recovered from a pediatric patient in a hospital from Buenos Aires (Argentina). By whole-genome sequencing, a total of 16 resistance genes were detected, including *bla*NDM-1 and mcr-1.5 (Faccone et al., 2019). In addition, coexistence of other multiple resistant determinants (e.g., *bla*VEB-3, *bla*TEM-1, and *bla*CMY-152) is reported (Yang et al., 2018) in *Citrobacter* spp.

Accumulating evidences of MDR pathogens suggest that it is imperative to explore novel and more efficacious alternatives for combating bacterial infections in the context of public health. Bacteriophages are ubiquitous bacterial viruses that occur in diverse environments, where they infect and lyze specific bacteria. Therefore, virulent bacteriophages have been exploited for various purposes such as aquaculture (Silva et al., 2014), food industry (Chibeu et al., 2012), agriculture (Svircev et al., 2018), and in medical applications (Fernández et al., 2018) to control the undesired bacterial populations. Nevertheless, the majority of these studies have focused on bacteriophages specific to *E. coli*, *Salmonella* spp., and *Vibrio* spp.; there is limited information on bacteriophages specific to *Citrobacter* spp. Furthermore, the majority of studies on bacteriophages specific to *Citrobacter* spp. have focused on *C. freundii* (Zhao et al., 2015; Hamdi et al., 2016). Recently, Mizuno et al. (2020) have isolated and characterized two new bacteriophages namely CrRp3 and CrRp10, which infect *C. rodentium*. Another recent study (Royam and Nachimuthu, 2020) from India has reported bacteriophages effective against *C. werkmanii* and *C. amalonaticus*. Nevertheless, there is meager information available on detailed phenotypic and genotypic characterization of bacteriophages of *C. amalonaticus.* This is despite several worldwide reports of UTI infections caused by *C. amalonaticus*. Consequently, the aim of the present study was to isolate a *C. amalonaticus* bacteriophage from sewage water, followed by detailed characterization and evaluation of the *in vitro* efficacy of the isolated bacteriophage against various bacterial species having public health and hygiene importance. In addition, we were interested in determining the storage stability and environmental stress tolerance of the isolated bacteriophage to generate data for making strategies with regard to subsequent field investigations. Moreover, the complete genome of the isolated bacteriophage was sequenced using Oxford Nanopore sequencing and the data were analyzed in detail using various bioinformatic approaches to elucidate its identity and biological properties.

## Materials and Methods

### Bacterial Strains

The sources of all bacterial strains used in this study are detailed in Table 1. Cultures were maintained on Luria–Bertani (LB) agar (1.5% agar w/v; BD Difco^TM^, United States) according to standard protocols.

**TABLE 1:** Host range and efficiency of plating (EOP) of bacteriophage HCF1.

**Bacterial species**	**Source**	Phage lyticpattern*	**Efficiency of plating**
*Citrobacter amalonaticus* NAIMCC 1364	^#^NAIMCC Mau, Uttar Pradesh, India	+	1.00
*Citrobacter freundii* NAIMCC 1351	^#^NAIMCC Mau, Uttar Pradesh, India	+	0.90
*E. coli* ATCC 25922	DRDE, Gwalior, India	–	–
*Shigella dysenteriae* type I	DRDE, Gwalior, India	–	–
*Salmonella typhi* 4736pg	DRDE, Gwalior, India	–	–
*Enterobacter clobae* NAIMCC 1255	^#^NAIMCC Mau, Uttar Pradesh, India	–	–
*Enterococcus faecium* NAIMCC 1045	^#^NAIMCC Mau, Uttar Pradesh, India	–	–
*Vibrio cholerae*	DRDE, Gwalior, India	–	–
*Staphylococcus aureus* ATCC25923	DRDE, Gwalior, India	–	–
*Klebsiella pneumoniae* MO2 (NCBI Accession No. MN387789)	DRDE, Gwalior, India	–	–
*Enterococcus saccharolyticus* NAIMCC 1332	^#^NAIMCC Mau, Uttar Pradesh, India	–	–
*Enterococcus faecium* NAIMCC 1456	^#^NAIMCC Mau, Uttar Pradesh, India	–	–

*Results recorded as +sensitive; –no infection.^#^National Agriculturally Important Microbial Culture Collection (NAIMCC).

### Bacteriophage Isolation

*Citrobacter amalonaticus* virus HCF1 was isolated from sewage water collected from the Defence Research and Development Establishment (DRDE) campus (26°19′ N, 78°17′ E), Gwalior, India. The sewage sample was centrifuged at 4000 × *g* for 10 min to eliminate possible solid impurities. The supernatant was then filtered using a 0.22-μm syringe filter (Millipex GP, Millipore, Cork, Ireland) to remove bacterial debris. The bacteriophage was isolated using the enrichment method described by Twest and Kropinski (2009). Briefly, 5 mL of exponentially growing *C. amalonaticus* NAIMCC 1364 culture (bacterial host) was inoculated into 90 mL of the sewage filtrate and incubated in a shaking incubator at 100 r/min for 24 h at 37°C. Following incubation, the mixture was centrifuged at 4000 × *g* for 10 min and filtered using 0.22-μm syringe filter (Millipex GP). Bacteriophage purification and propagation was carried out using double agar plating assay as described by Adams (1959).

### Electron Microscopy of Bacteriophage

To prepare bacteriophage sample for the morphological examination using transmission electron microscope (TEM), the purified bacteriophage solution (10 μL, 10^11^ PFU mL^–1^) was deposited on a 300-mesh copper grid and stained with 2% phosphotungstic acid (pH 4.5) for 30 s. After air-drying, grid was observed with a TEM (JEOL JEM-1400plus) at an accelerated voltage of 80 kV. Three virions were measured to determine the size of head and tail. Accordingly, the values have been represented in results as average ± standard deviation (SD).

### One-Step Growth Curve Analysis

The one-step growth experiment was performed as described by Hyman and Abedon (2009). Briefly, bacteriophages were added into the exponentially growing *C. amalonaticus* at an MOI of 0.1 and incubated at 37°C for 5 min. This admixture was centrifuged at 10,000 × *g* for 2 min to eliminate non-adsorbed bacteriophages. The pellet was resuspended in 10 mL of LB broth and incubated at 37°C for 100 min. From this, 100 μL of sample was drawn at 10 min interval, and bacteriophage titer, burst size, and latent period were determined using the double agar plating assay as described earlier (Adams, 1959).

### Host Range and Efficiency of Plating

Eleven bacterial species commonly found in sewage effluent and having public health concern were selected in this study (Table 1). Host range and efficiency of plating (EOP) of isolated bacteriophage HCF1 were investigated against these bacterial species using the method suggested by Kutter (2009). Briefly, 0.5 mL of the test strain grown overnight in LB broth was taken and mixed with 4.5 mL of soft agar (0.6% agar w/v; BD Difco^TM^). This mixture was plated onto LB agar plate (1.5% agar w/v; BD Difco^TM^). Subsequently, 10 μL of bacteriophage suspension (∼10^10^ PFU mL^–1^) was spotted onto the LB agar medium and allowed to incubate overnight at 37°C. Each experiment was performed in triplicate. The relative EOP with respect to various susceptible bacterial hosts was determined using the double agar plating assay as described previously (Adams, 1959).

### Storage Stability

Bacteriophages were stored at 25 and 4°C in SM buffer (NaCl 100 mM, MgSO_4_⋅7H_2_O 8 mM, Tris-Cl 50 mM of pH 7.5), as well as at −20 and −80°C in SM buffer supplemented with 15% (v/v) glycerol in multiple pre-sterilized glass vials (10 mL volume). Samples were removed from the glass vials at a 15-day (D) interval and the bacteriophage titer was determined using the double agar plating assay as described earlier (Adams, 1959).

### Stress Tolerance

The thermal stability, pH stability, and salt tolerance of the isolated bacteriophages were tested at various temperatures (35, 40, 45, and 50°C), pH (5.0, 7.0, 9.0, and 11.0), and NaCl concentrations (5, 10, 15, and 20 g L^–1^). Acidity and alkalinity in solutions were produced by, respectively, using HCl (6 M) and NaOH (6 M) as described by Niu et al. (2012). Next, 10 mL of bacteriophage suspension (∼10^8^ PFU mL^–1^) was added to 90 mL of autoclaved SM buffer with varying pH and salt concentrations and incubated at 37°C in conical flask (250 mL volume) made of borosilicate glass. Samples were drawn from the flasks 30, 60, 120, 240, and 360 min after incubation, and the bacteriophage titer was determined using the double agar plating assay as described previously (Adams, 1959).

### *In vitro* Efficacy Assay

*In vitro* efficacy testing was performed in autoclaved saline buffer (0.9% NaCl; pH 7.0) and in an anaerobic biodigester effluent at 37°C. Effluent was obtained from a local anaerobic biodigester that treats human excreta. Briefly, 10 mL of bacterial culture (10^9^ CFU mL^–1^) was added to 89 mL of autoclaved saline buffer/effluent in a conical flask (Borosilicate glass; 250 mL volume) which resulted in 10^8^ CFU mL^–1^ of bacterial suspension. One milliliter each of bacteriophage suspension having 10^10^, 10^11^, and 10^12^ PFU mL^–1^ was used to make three different MOIs (1, 10, and 100), and the flasks were incubated at 37°C. Next, 100 μL of the sample was drawn at 1 h intervals up to 6 and at 24 h. CFU counts were performed using the Miles and Mishra drop-plate method (Miles et al., 1938). In the process, 100 μL of the sample was serially diluted in NaCl (0.9%), and then 20 μL of each dilution was dropped onto the surface of the LB agar. The drop was allowed to spread naturally. Plates were incubated for 18 h at 37°C. Following incubation, bacterial colonies were counted and CFU was calculated using the formula provided below:

CFU mL^–1^ = Average number of colonies for a dilution × 50 × dilution factor.

### Bacteriophage Nucleic Acid Extraction

Bacteriophage HCF1 nucleic acid extraction was performed as described by Pickard (2009) with some modifications. One milliliter of the bacteriophage suspension (10^9^ PFU mL^–1^) was incubated with DNase (2 mg mL^–1^) and RNase (5 mg mL^–1^) for 1 h at 37°C. To this, bacteriophage lysis buffer (100 μL of 10% SDS, 50 μL of 0.5 M EDTA, and 10 μL of 10 mg mL^–1^ proteinase K; pH 7.0) was added, mixed well, and incubated at 50°C for 30 min. For protein precipitation, 3.5 M ammonium acetate was added to this mixture at the rate of 60 μL mL^–1^, incubated on ice for 30 min, and centrifuged at 13,000 × *g* for 10 min. Thereafter, the supernatant was taken into a fresh tube, where an equal volume of phenol: chloroform: isoamyl alcohol (25:24:1) was added, and the sample was centrifuged at 10,000 × *g* for 10 min. This step was repeated once. The aqueous upper phase was then transferred into a fresh tube, and an equal volume of chloroform was added to it before the sample was centrifuged at 10,000 × *g* for 10 min. The aqueous upper phase was again transferred to a new tube and an equal volume of isopropanol was added and the sample was allowed to precipitate at 20°C for 2 h. The precipitated nucleic acid was centrifuged at 12,000 × *g* for 5 min at 4°C, and the pelleted nucleic acid was washed with 70% (v/v) ethanol. The purified bacteriophage nucleic acid was visualized on a 0.9% (w/v) agarose gel. The nucleic acid was distinguished as being DNA or RNA following treatment with DNAse I and RNAse A (Fermentas) separately as per the manufacturer’s instructions.

### Library Preparation and Nanopore Sequencing

End-repairing of bacteriophage HCF1 DNA samples was done using NEBnext ultra II end repair kit (New England Biolabs, Ipswich, MA, United States). Clean-up of end-repaired samples was carried out with 1x AmPure beads (Beckmann Coulter, United States). NEB blunt/TA ligase (New England Biolabs) using NBD103 (ONT) was used for Native barcode ligation. Following ligation, clean-up process was repeated with 1x AmPure beads (Beckmann Coulter). The Qubit-quantified barcode ligated DNA sample was then pooled at equimolar concentrations to obtain 500 ng pooled sample. BAM Adapter ligation was performed for 15 min using NEBnext Quick Ligation Module (New England Biolabs). The library mix was cleaned up using 0.4X AmPure beads (Beckmann Coulter) and, finally, the sequencing library was eluted in 15 μL of elution buffer and used for Nanopore sequencing. Sequencing was performed on a GridION X5 (Oxford Nanopore Technologies, Oxford, United Kingdom) using a SpotON flow cell (R9.4) in a 48 h sequencing protocol on MinKNOW 2.1 v18.05.5. In order to eliminate probable errors in long-read assemblies, all the Nanopore raw reads (“*fast5*” format) were basecalled (“*fastq5*” format) and demultiplexed using Albacore v2.3.1. Basecalled reads were error-corrected and assembled using “Canu” assembler v1.8, for sequence polishing.

### Bioinformatic Analysis

The assembled bacteriophage HCF1genome was analyzed using RASTtk^1^ (Brettin et al., 2015) with customized RASTtk pipeline call features glimmer3, prodigal, and genemark for predicting putative open reading frames (ORFs). Annotation was carried out using RAST annotation scheme by enabling the “annotate protein” option in the program. Functional prediction of ORFs was confirmed by BLASTp^2^ (Altschul et al., 1997) search with database of non-redundant protein (nr) sequences, Pfam^3^ (Finn et al., 2016), InterProScan^4^ (Mitchell et al., 2014), and the VirFam server^5^ (Lopes et al., 2014). Transmembrane domains were predicted using TMHMM v.2^6^ (Krogh et al., 2001), and the genomic map was visualized using CGview software^7^ (Grant et al., 2012). Putative tRNAs were identified using tRNAscan- SE 1.21^8^ (Lowe and Chan, 2016). The Phyre2 server was used for the prediction and analysis of protein structures and functions (Kelley et al., 2015). For pairwise comparisons of the whole-genome nucleotide sequences, the Genome-BLAST Distance Phylogeny (GBDP) method (Kolthoff et al., 2013) was used under the suggested settings for prokaryotic viruses (Kolthoff and Göker, 2017), in the VICTOR program^9^. The phylogenomic GBDP trees were inferred using the formulae D0, D4, and D6 which yielded average support of 5, 24, and 10%, respectively. Accordingly, the phylogenomic tree with D4 formula providing maximum average support has been taken into consideration and depicted in Figure 5A.

Phylogenetic analysis based on individual genes (capsid and scaffold protein, and portal protein) was carried out using the GGDC web server (Kolthoff et al., 2013) with the DSMZ phylogenomics pipeline (Krogh et al., 2001) adapted to individual genes. For maximum-likelihood (ML) analysis, rapid bootstrapping combined with the auto MRE bootstopping criterion (Pattengale et al., 2010) and subsequent search for the best tree was used. For maximum parsimony (MP) analysis, 1000 bootstrapping replicates were used in combination with tree-bisection-and-reconnection branch swapping and 10 random sequence addition replicates. Subsequently, the best amino-acid substitution matrix (with empirical frequencies) was determined with RAxML using an ML starting tree. The percent sequence similarity matrix was generated using the Clustal Omega v2.1 tool^10^ for whole-genome and protein sequences (David, 2018). Furthermore, in order to determine the core sets of genes and to evaluate the protein homologies of HCF1 with other related viruses, seven bacteriophages exhibiting maximum similarity as suggested through Clustal Omega were selected, and their corresponding protein data were subjected to core gene analysis using CoreGenes v3.5 with default threshold setting of 75 (Turner et al., 2013). In addition, EasyFig genome visualizer was used for comparatively analyzing various modules (Sullivan et al., 2011).

### Accession Numbers

The complete genome sequence of *C. amalonaticus* virus HCF1 has been deposited in NCBI GenBank under accession number MN545971. In addition, *fastq* file pertaining to raw sequence data has been submitted to NCBI Sequence Read Archive (SRA) database under the identifier number SRP267319.

### Statistical Analysis

Statistical analysis for the empirical data was performed using SPSS Statistics v17.0 (SPSS Inc., Chicago, IL, United States). Significant differences among the different conditions for each tested parameter were assessed by one-way analysis of variance (ANOVA) followed by Tukey’s HSD *post hoc* test. A *p-*value ≤ 0.05 was considered to be statistically significant. Accordingly, significant differences have been marked in tables and figures as relevant.

## Results and Discussion

### Bacteriophage Life Cycle, Host Lytic Spectrum, and *in vitro* Efficacy

**FIGURE 1:**
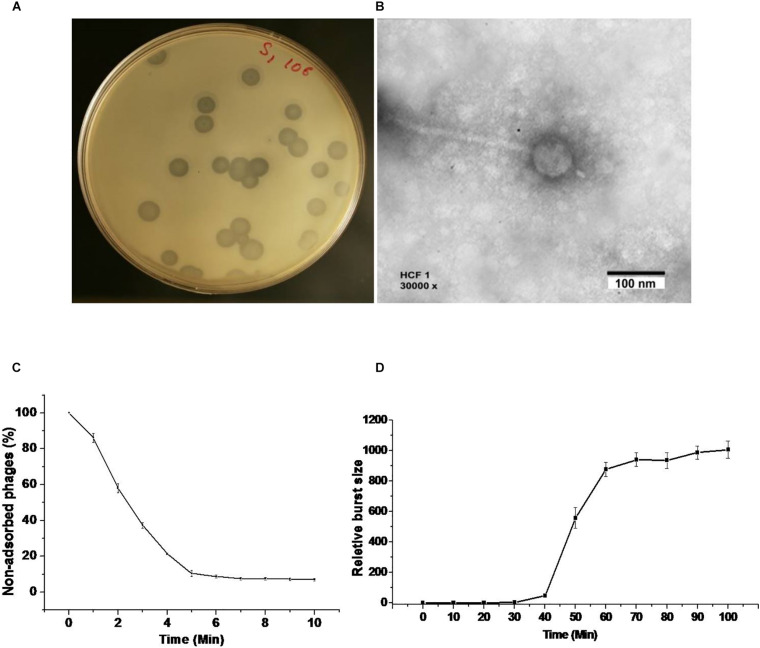
Morphology, adsorption rate, and one-step growth curve of bacteriophage HCF1. **(A)** Bacteriophage plaque in double agar plating assay. **(B)** Transmission electron micrograph. **(C)** The rate of adsorption of bacteriophage to the *C. amalonaticus* NAIMCC 1364 host. Bacteriophages were added to the bacterial suspension at an MOI:0.1. The percentage of non-adsorbed bacteriophages was calculated at the indicated time points. The presented data are means of three independent experiments with error bars showing the standard error of mean (SEM). **(D)** One-step growth curve analysis of bacteriophage HCF1. Plaque-forming units (PFU) were estimated at different intervals post infection against host bacterium *C. amalonaticus*. Bars represent SEM.

We were able to get bacteriophage number as high as 10^12^ PFU mL^–1^ by following the classical method for purification and propagation as suggested by Adams (1959). The bacteriophage HCF1 formed conspicuous plaques with a diameter of about 4 mm (Figure 1A). It has an icosahedral head of 66 ± 6 nm dia (*n* = 3; ± SD) and a tail of 233 ± 4 nm dia (*n* = 3; ± SD) as analyzed using TEM (Figure 1B). More than 80% of the bacteriophages adsorbed to the host within 6 min (Figure 1C). One-step growth curve analysis, as studied by infecting the *C. amalonaticus* NAIMCC 1364 with the bacteriophage, suggests that HCF1 has a latent period of 30 min and that the estimated burst size is 121 PFU per bacterial cell (Figure 1D). The literature suggests that the latent period and burst size of other *Citrobacter* spp. bacteriophages are 20–25 min and 45–801 PFU per bacterial cell, respectively (Chaudhry et al., 2014; Oliveira et al., 2016) which agrees well to the present observations. Host lytic spectrum determination of HCF1 against different bacterial species by spot testing shows that the bacteriophage could infect *C. freundii* and *C. amalonaticus* both. Additionally, bacteriophage infectivity, as ascertained further through EOP, was found to vary slightly with different species of *Citrobacter* (Table 1).

*In vitro* efficacy data showed a statistically significant decrease (1.23 log) in bacterial counts within 1 h of bacteriophage treatment even at an MOI:1 compared to the control (*p* ≤ 0.05 as per Tukey’s HSD test). Data recorded after 1 h suggested that an increase in the MOI resulted in a significant decrease in bacterial growth. HCF1 completely eliminated the host within 4 and 5 h at MOI:100 and MOI:10, respectively. Even at MOI:1, there was 6.05 log reduction in bacterial counts within 6 h compared to the control (Table 2). Interestingly, bacteriophage efficacy did not differ much with different treatment media (i.e., saline solution or Biodigester effluent). In literature, detailed studies on bacteriophages of *C. amalonaticus* are meager. In a study (Oliveira et al., 2016) evaluating antibacterial effect of bacteriophage CfP1 on *C. freundii*, it was found that CfP1 proved to be efficient to reduce *Citrobacter* populations to residual numbers up to 8-h incubation period at MOI:10. Variation in the efficacy results implies that the MOI is an important factor as it gives an idea of the optimal dose required for combating *Citrobacter* infections.

**TABLE 2:** *In vitro* assay of bacteriophage HCF1 against *Citrobacter amalonaticus* in saline solution and in an anaerobic biodigester effluent.

**Conditions**	**Time**	**Control**	**MOI 1**	**MOI 10**	**MOI 100**
Saline	0h	8.80 ± 0.04a	8.46 ± 0.09a	8.64 ± 0.07a	8.54 ± 0.27a
	1h	8.76 ± 0.04a	7.53 ± 0.12b	7.65 ± 0.09b	6.53 ± 0.07c
	2h	8.72 ± 0.05a	6.48 ± 0.09b	5.35 ± 0.19c	4.52 ± 0.14d
	3h	8.63 ± 0.06a	5.28 ± 0.20b	4.28 ± 0.20c	2.51 ± 0.13d
	4h	8.55 ± 0.08a	4.59 ± 0.06b	3.45 ± 0.23c	0.00*d
	5h	8.44 ± 0.07a	3.67 ± 0.04b	0.00*c	0.00*c
	6h	8.24 ± 0.16a	2.19 ± 0.12b	0.00*c	0.00*c
	24h	7.53 ± 0.12a	0.00*b	0.00*b	0.00*b
Anaerobic biodigester effluent	0h	8.89 ± 0.06a	8.74 ± 0.14a	8.42 ± 0.25a	8.67 ± 0.11a
	1h	8.81 ± 0.11a	7.57 ± 0.09b	7.67 ± 0.11b	6.51 ± 0.17c
	2h	8.74 ± 0.07a	6.66 ± 0.07b	5.65 ± 0.09c	3.65 ± 0.12d
	3h	8.68 ± 0.10a	4.57 ± 0.14b	3.65 ± 0.03c	2.42 ± 0.21d
	4h	8.61 ± 0.22a	3.62 ± 0.09b	2.73 ± 0.10c	0.00*d
	5h	8.67 ± 0.04a	2.79 ± 0.10b	0.00*c	0.00*c
	6h	8.26 ± 0.18a	2.23 ± 0.12b	0.00*c	0.00*c
	24h	9.46 ± 0.14a	0.00*b	0.00*b	0.00*b

*No growth was observed; log-transformed CFU values are presented in the table. ± Standard error of mean (*n* = 3). Values of a given bacteriophage depicted in column without common letter differ significantly at *p* ≤ 0.05 as per Tukey’s HSD test.

### Storage Stability

**FIGURE 2:**
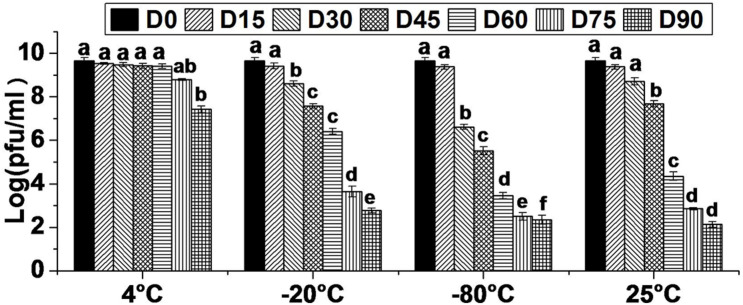
Viability of bacteriophage HCF1 stored at varying temperature regimes. Error bar represents SEM (*n* = 3). Values of a given bacteriophage depicted as column without common letter differ significantly at *p* ≤ 0.05 as per Tukey’s HSD test. Letter “D” represents the experimental duration in days.

The storage stability study was conducted with a view to collect the data on effect of varying temperature regimes (4, −20, −80, and 25°C) on viability of bacteriophage HCF1. The results revealed that bacteriophage viability did not decrease significantly for up to 60 D (*p* > 0.05 as per Tukey’s HSD test) at 4°C. Even at 75 and 90 D, bacteriophage survival was as high as 8.80 and 7.43 log PFU mL^–1^. In contrast, when stored at −20 and −80°C, bacteriophage viability decreased significantly over time from 15 D onward (*p* < 0.05 as per Tukey’s HSD test) and, at 90 D, only 2.77 and 2.34 log PFU mL^–1^ survival was recorded, respectively. There was no statistically significant decrease in bacteriophage survival following storage at 25°C, for up to 30 D (*p* > 0.05 as per Tukey’s HSD test). However, after 30 D, bacteriophage titer decreased significantly over time with the survival of only 2.14 log PFU mL^–1^ at 90 D (Figure 2). Generally, long-term stability varies with different bacteriophages, thereby necessitating the determination of optimal storage conditions for a newly isolated bacteriophage having biocontrol potential. Comparative storage stability data of bacteriophage HCF1 suggested 4°C to be the most suitable temperature for its preservation. In general, the tailed bacteriophages have been reported to remain active for 6 months (Chaudhry et al., 2014) and even up to 12 months (Merabishvili et al., 2009) when stored at 4°C. However, differences in sensitivity of individual bacteriophages to storage conditions and the composition of the storage media are often observed (Ackermann et al., 2004; Fortier and Moineau, 2009) which necessitates the generation of data in respect of newly isolated bacteriophage. In the present study, storage of bacteriophage HCF1 at freezing temperatures (with glycerol at −20 and −80°C) resulted in loss of titer as high as 6.90 log and 7.33 log, respectively (Figure 2). It has been suggested by Warren and Hatch (1969) that the crystal structure of ice may cause destruction of bacteriophages while storage at −20°C, which may explain the loss of titer as observed in the present study. Olson et al. (2004) recommended that bacteriophages should be maintained at −80°C to protect them from inactivation during storage beyond 40 D. However, the results of present study did not corroborate this. Furthermore, in the present study, the addition of glycerol as a cryopreservative agent at freezing temperatures did not improve the stability of HCF1 (Figure 2). Similar to the present findings, previous studies have shown that storage at 4°C results in improved stability compared to freezing with glycerol (Clark and Geary, 1973; Alvi et al., 2018).

### Environmental Stress Tolerance

**FIGURE 3:**
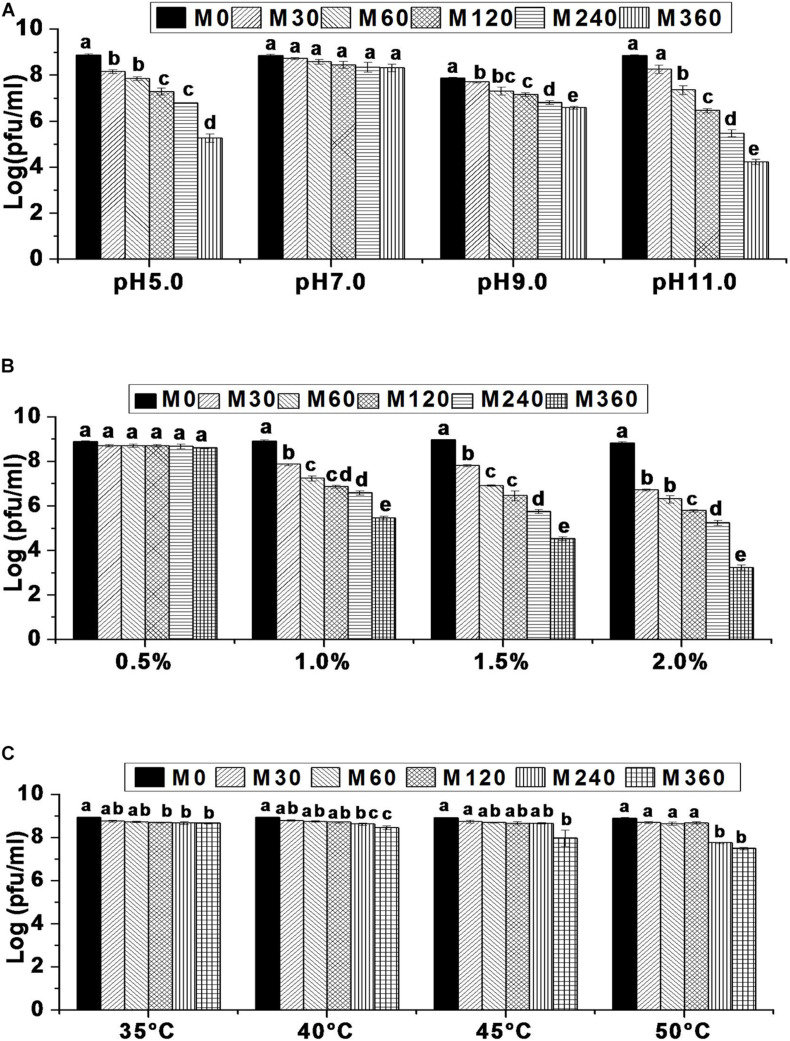
Environmental stress tolerance of bacteriophage HCF1. **(A)** pH stability. **(B)** NaCl tolerance. **(C)** Thermal stability. Error bars represent standard error of mean (*n* = 3). Values of a given bacteriophage depicted as column without common letter differ significantly at *p* ≤ 0.05 as per Tukey’s HSD test. Letter “M” represents the experimental duration in minutes.

Environmental factors such as temperature, pH, and salinity are known to influence the rate of bacteriophage adsorption and intracellular replication (Leverentz et al., 2001) which, in turn, affects phage survival (Srinivasan et al., 2007; Chaudhry et al., 2014; Oliveira et al., 2016). Hence, it is important to evaluate bacteriophage tolerance to environmental variables before they are used in different applications. In this work, it has been shown that the survival rate of bacteriophage HCF1 is 5.26, 6.81, and 4.24 log at pH 5.0, pH 9.0, and pH 11.0, respectively, indicating considerable tolerance to the environmental extremes. Although highly acidic and alkaline conditions negatively influenced HCF1 viability, at pH 5.0 and pH 11.0, survival rates for HCF1 were 5.26 log and 4.24 log, respectively, compared to the control. At pH 5.0, there was no significant difference in bacteriophage survival between 120 and 240 min (*p* > 0.05 as per Tukey’s HSD test). Bacteriophage HCF1 tolerated pH 11.0 for up to 30 min without any significant decrease in viability. At pH 9.0, bacteriophage HCF1 demonstrated increased tolerance toward alkaline conditions as shown by the considerable phage survival (6.81 log) for up to 360 min. However, bacteriophage HCF1 showed no statistically significant decrease in viability during the tested period at pH 7.0 for up to 360 min (*p* > 0.05 as per Tukey’s HSD test; Figure 3A). Another study (Oliveira et al., 2016) observed no considerable loss of phages in the range of 5–11 even after 24 h.

In terms of salt tolerance, bacteriophage viability decreased as salinity increased over a period of time with varying degrees of sensitivity. It was found that 5 g NaCl L^–1^, was well-tolerated by bacteriophage HCF1 with no statistically significant difference (*p* > 0.05 as per Tukey’s HSD test) in its viability over the studied time compared to the control. In contrast, we observed moderate sensitivity of bacteriophage HCF1 to 10 g NaCl L^–1^ (survival 5.46 log). However, further increase in salinity to 15 and 20 g NaCl L^–1^ resulted in decreased viability with survival rates of 4.53 and 3.24 log, respectively (Figure 3B). Here, the evaluation was carried out using magnesium-supplemented SM buffer, which is known to enhance electrostatic bonding, thereby facilitating bacterial attachment (Moldovan et al., 2007). Although, at low concentrations, salt could facilitate the infection and growth of bacteriophages (Baross et al., 1978), at higher concentrations, salt can adversely affect the structural stability of the bacteriophage proteins/nucleic acid (Hippel and Schleich, 1969) which could explain the decreased viability of bacteriophage HCF1 with elevated salt levels. Moreover, the present data show that bacteriophage viability decreased with increased salinity with varying degrees of sensitivity. It was observed that 5 g NaCl L^–1^ was well-tolerated by bacteriophage HCF1 while 20 g NaCl L^–1^ resulted in decreased viability with a survival rate of 3.24 log. Somewhat similar to the present observations, Silva (2005) found that bacteriophages retained activity in salt concentrations from 6–10 g L^–1^ even by D 52.

Thermal stability data suggest that bacteriophage HCF1 tolerates relatively high temperatures very well. Even at 50°C, there was no statistically significant decrease in its viability up to 120 min compared to the control (*p* > 0.05 as per Tukey’s HSD test). The bacteriophage survival rate at 50°C after 360 min was 7.50 log (Figure 3C). Previous studies have shown that *C. freundii* phage LK1 was stable at 50°C for up to 1 h (Chaudhry et al., 2014) while *C. freundii* phage CfP1 was stable at 50°C for up to 24 h (Oliveira et al., 2016).

### Bacteriophage Genome Analyses

#### Genome Characteristics of HCF1

**FIGURE 4:**
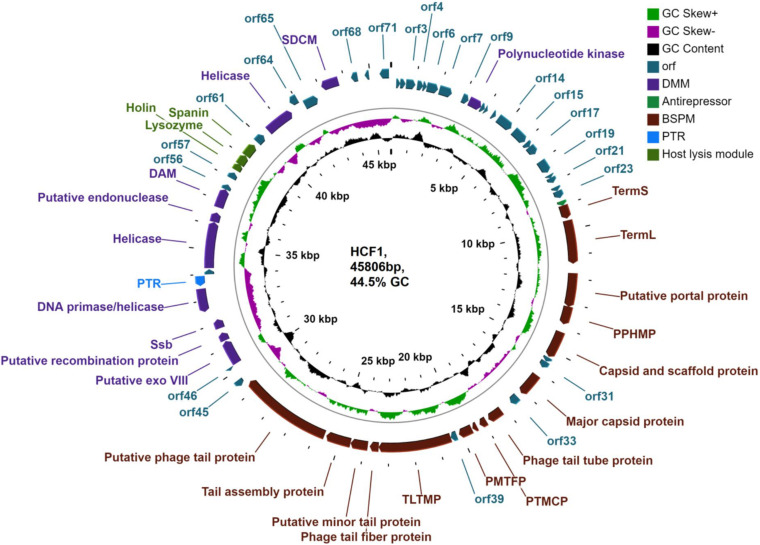
Genome organization of *Citrobacter* virus HCF1. ORFs with putative annotations are represented by specific colors as per their functional categories marked using arrows. PTMCP, putative tape measure chaperone protein; PPHMP, putative phage head morphogenesis protein; Ssb, single-strand DNA binding protein; SDCM, site-specific DNA-cytosine methylase; TermS, small terminase subunit; TermL, large terminase subunit; PMTFP, putative minor tail fiber protein; TLTMP, tail length tape-measure protein; PTR, putative transcriptional regulator; Putative exo VIII, putative exodeoxyribonuclease VIII; DAM, DNA adenine methyltransferase; BSPM, bacteriophage structure and packaging module; DMM, DNA metabolism module.

DNAse I treatment of the nucleic acid belonging to bacteriophage HCF1 led to digestion of the bacteriophage genome whereas RNAse A treatment had no effect on it, thereby suggesting that HCF1 is a DNA virus. Furthermore, the analysis results revealed that the HCF1 genome has a double-stranded linear DNA molecule of 45.8 kbp size with a GC content of 44.5%. However, tRNA gene was not found in the HCF1 genome, thereby implying its dependence on host tRNA for protein synthesis. Based on RAST analysis, 71 putative ORFs were identified, out of which functions of 30 ORFs were predicted with the help of BLASTp analysis. Functions to the remaining 41 ORFs could not be ascribed (Figure 4 and Supplementary Table S1). The functional proteins of the bacteriophage HCF1 belonged to three modules: a host lysis module, a phage structure and packaging module, and a DNA metabolism module. In addition, an antirepressor (encoded by *orf*24) and putative transcriptional regulator (encoded by *orf*51) with 68 and 46% sequence similarity to antirepressors of *Salmonella* spp. phage 36 and putative transcriptional regulator of *Escherichia* spp. phage vB_EcoS_W011D, respectively, were identified (Figure 4, Supplementary Figure S1, and Supplementary Table S1). Putative antirepressor and transcriptional regulator in bacteriophage HCF1 genome indicates the presence of a mechanism for decrease in the transcription of early genes and increase in the transcription of late genes. Recently, it has been established that late gene activation promotes the lytic pathway and the early gene activation plays a role in active lysogeny (Pasechnek et al., 2020). In addition, no ORFs were identified corresponding to excisionase, integrase, or repressor genes in the genome of HCF1. Based on these observations, it is hypothesized that the bacteriophage HCF1 has a lytic lifecycle. Detailed genome analysis suggested that the novel bacteriophage HCF1 belongs to the subfamily *Tempevirinae* of the *Drexlerviridae* family. To the best of our knowledge, only three *Citrobacter* spp. baceriophages belonging to the *Drexlerviridae* family have been described in detail so far (i.e., NCBI Nos. NC_027350, Shaw et al., 2015; KY694971, Kim et al., 2018; MH729819, Sun et al., 2009).

#### Host Lysis Module

The host lysis module was restricted to *orf*58, *orf*59, and *orf*60, whose protein products had 53.52, 54.79, and 90.38% sequence similarity to holin of *Salmonella* spp. phage vB_SenS_PHB07, lysozyme of *Escherichia* spp. phage vB_EcoS_PHB17, and to unimolecular spanin protein of *Escherichia* spp. phage LL5, respectively, as per BLASTp analysis (Figure 4 and Supplementary Table S1). In addition, the lysis cassette module of HCF1 was found to be arranged in a conserved order: holin (38,363–38,662 bp), lysozyme (38,634–39,071 bp), and spanin (39,068–39,613 bp) (Supplementary Figure S1).

Furthermore, TMHMM2.0 analysis of the lysis module suggests that the proteins corresponding to *orf*58 and *orf*60 have an N-terminal signal sequence and a transmembrane helix (sequence position 25–47 for *orf*58 and 10–32 for *orf*60). TMHMM2.0 analysis could not provide useful information regarding *orf*59. Hence, Pfam analysis of *orf*59 was carried out which suggested the presence of a transmembrane domain with a signal peptide (E-value—3.5e-15) in the bacteriophage lysozyme, indicating a likely function of O-glycosyl hydrolases (EC 3.2.1.17). Phyre2 server analysis suggested that *orf*59 corresponding to the HCF1 lysozyme had a maximum identity to coliphage 21 lysozyme (confidence level 100%; identity 41%; residues 8–143; Supplementary Figure S2). Lysozymes are known to be first secreted in an inactive form while anchored to the membrane by the N-terminal signal anchor release (SAR) domain (Park et al., 2006). This feature plays an important role in preventing premature lysis of the infected host cell (Casjens and Hendrix, 1974). The predicted presence of lysozyme in bacteriophage HCF1 suggests that it facilitates the prevention of premature host lysis.

#### Bacteriophage Structure and Packaging Module

BLASTp analysis identified 15 ORFs related to the bacteriophage structure and packaging module (BSPM; Supplementary Table S1). Further analysis of conserved proteins of the BSPM using VirFam server, which is exclusively used for the identification of head, neck, and tail proteins, indicated that the proteins related to *orf*33 and *orf*34 were also part of this module. These proteins were identified by BLASTp analysis as hypothetical protein (*orf*33) and gp41 (*orf*34), and were found to encode for neck protein Type 1 (Ne1) and Tail completion protein Type 1 (Tc1), respectively (Supplementary Figure S3 and Supplementary Table S2). These proteins exhibited similarity to those of *Escherichia* spp. phage TLS with 88 and 61% sequence identity, respectively (Supplementary Table S2). Interestingly, analysis of the conserved BSPM proteins using the VirFam server suggests the presence of an Ne1 superfamily gene in the HCF1 neck module. A recent description of the Ne1 gene superfamily has shown that Ne1 proteins show a considerable degree of versatility in terms of size and may range from 56 to 231 residues (Lopes et al., 2014). Ne1 has a crucial role in virion head-to-tail connection assembly and infectivity. One study reported that when Ne1 protein GpZ is not available, viral particles are still produced but they exhibit low infectivity (Auer and Schweiger, 1984). Therefore, the presence of Ne1 proteins in bacteriophage HCF1 indicates its potential infectivity as also evident by the results of efficacy determination.

#### DNA Metabolism Module

BLASTp analysis suggests that the DNA metabolism module consists of 10 ORFs (*orf*10, ORFs 47–50, ORFs 53–55, *orf*63, and *orf*66). Out of these, *orf*53 was the largest ORF (1860 bp, 619 aa) encoding a protein similar to the ATP-dependent DNA helicase of *Citrobacter* virus Stevie sharing 59.97% identity (accession number YP_009148736.1). The predicted proteins of *orf*10, *orf*47, *orf*48, *orf*49, *orf*50, *orf*54, and *orf*63 displayed 74.01, 61.67, 82.22, 81.13, 57.30, and 97.46% identity to polynucleotide kinase, putative exodeoxyribonuclease VIII, putative recombination protein, Ssb, DNA primase/helicase, putative endonuclease, and helicase with *Escherichia* phage vB_EcoS_PHB17, *Salmonella* phage YSP2, *Salmonella* phage 36, *Escherichia* virus TLS, *Citrobacter* phage CF1 DK-2017, *Salmonella* phage GJL01, and *Salmonella* phage 36, respectively (Supplementary Table S1 and Figure 4). Interestingly, Exodeoxyribonuclease VIII breaks double-stranded DNA and degrades a genome on both 5′ ends (Sharma et al., 2019), thereby allowing the kinked and abnormal portions of a genome to be straightened and repaired through homologous recombination. Furthermore, exodeoxyribonuclease VIII facilitates repair of the genome even in low-energy milieu as it does not require ATP to perform DNA repair (Sharma et al., 2019). It is therefore hypothesized that exodeoxyribonuclease VIII enables the bacteriophages to remain stable despite mutations from UV damage (Sharma et al., 2019). Predicted presence of exodeoxyribonuclease VIII (*orf*47) in HCF1 genome may confer it stability against DNA damage.

Phyre2 server analysis suggested that *orf*59 corresponding to the bacteriophage HCF1 putative endonuclease had a structural and functional similarity with a viral type replication and repair nuclease (VRR-Nuc) (confidence level 99.81%; identity 30%; residues 56–130; PDB entry: c4qblF). VRR-Nuc is known to be member of the primordial restriction endonuclease-like superfamily possessing a mixed fold of α/β fold of αβββαβ topology. VRR-Nuc-containing proteins usually occur as a single-domain nuclease in several bacteria and viruses. FANCD2/FANCI-associated nuclease 1 (FAN1), a structure-specific nuclease, is the only example of multi-domain eukaryotic protein containing a VRR-Nuc domain. FAN1 is considered to play an important role in the repair of inter-strand DNA crosslinks like the ERCC1-XPF nuclease (DuPrez et al., 2020).

Furthermore, prediction of transmembrane domains using TMHMM2.0 analysis revealed that *orf*66 (site-specific DNA-cytosine methylase) contained an N-terminal signal sequence which lacked any transmembrane domains. Pfam analysis suggests that the protein corresponding to *orf*50 in bacteriophage HCF1 consists of a zinc-binding domain (PF08273, E-value—9.7e-13), a transmembrane domain, a signal peptide, and a coiled-coil. In addition, the protein corresponding to *orf*53 in HCF1 contains a helicase conserved C-terminal domain restricted to DEAD/H helicases (PF00271, E-value—2.7e-06), a transmembrane domain, and a signal peptide. Furthermore, the predicted protein of *orf*55 of HCF1 exhibited 64.23% identity with DNA adenine methyltransferase (DAM; E-value 7e-104; accession number AVQ09766.1) of *Salmonella* phage vB_SenS_PHB07. DAM is linked with several biological functions in bacteria (e.g., as an epigenetic switch, particularly with virulence gene regulation). In addition, DAM is known to induce DNA methylation in T1 bacteriophages (Bochow et al., 2012). This feature facilitates protection of bacteriophage DNA against the restriction modification (R-M) system of the host bacteria. More recently, it has also been hypothesized that DAM plays a central role in the lysogenic and lytic states of bacteriophage (Bochow et al., 2012). The predicted occurrence of DAM in HCF1 suggests that it is able to evade the host R-M system.

### New Member Assigned to the *Drexlerviridae*

**FIGURE 5:**
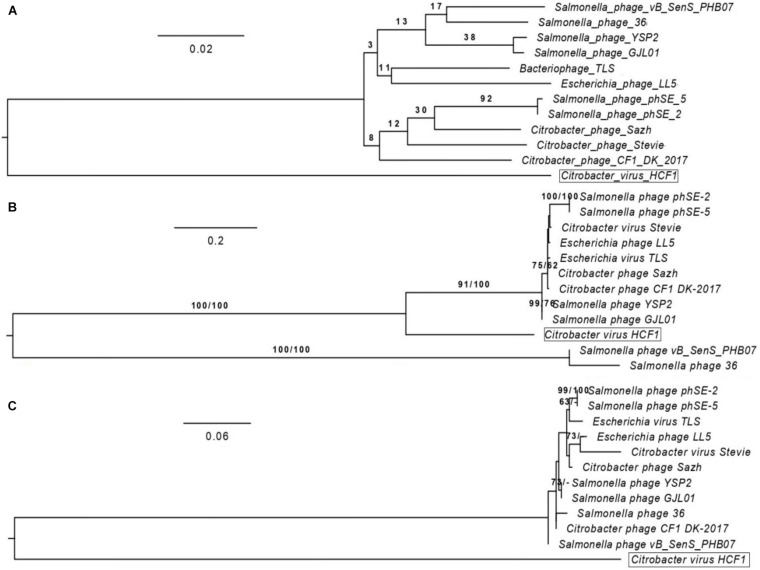
Phylogenomic comparison of bacteriophage HCF1 with 11 closely related members of *Drexlerviridae*. The numbers above branches are pseudo-bootstrap support values from 100 replications. The branch lengths of the resulting VICTOR trees are scaled in terms of the respective distance formula used. **(A)** Phylogenomic Genome-BLAST Distance Phylogeny (GBDP) tree generated using VICTOR based on whole-genome analysis. **(B)** Phylogeny tree generated through GGDC web server using the DSMZ phylogeny pipeline adapted to individual gene (capsid and scaffold protein). Maximum-likelihood (ML) tree was inferred under the VT + GAMMA model and rooted by midpoint-rooting. The branches are scaled in terms of the expected number of substitutions per site. The numbers above the branches are support values when larger than 60% from ML (left) and MP (right) bootstrapping. **(C)** Phylogeny tree generated through GGDC web server using the DSMZ phylogeny pipeline adapted to individual gene (Portal protein). Maximum-likelihood (ML) tree was inferred under the JTT + GAMMA model with other settings kept same as depicted for capsid and scaffold protein (Figure 5B).

**FIGURE 6:**
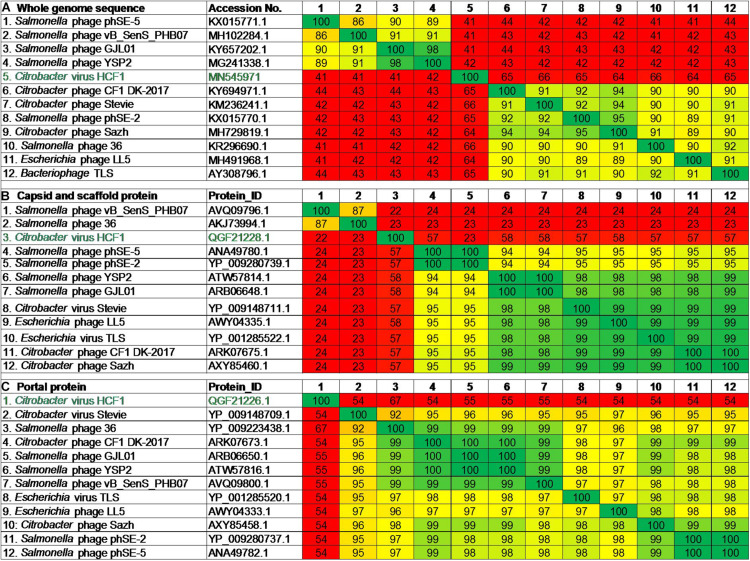
Percentage similarity matrix of bacteriophage HCF1 along with 11 closely related members of *Drexlerviridae* generated using Clustal Omega from the data pertaining to **(A)** whole-genome sequence, **(B)** capsid and scaffold protein, and **(C)** portal protein.

BLASTn analysis of the whole-genome sequence of the newly isolated bacteriophage HCF1 suggested that only 11 bacteriophages had an E-value of 0.0. These 11 bacteriophages were taken forward for detailed comparison with HCF1. Maximum score, query cover, and percent identity of these bacteriophages with HCF1 ranged from 974 to 1037, 15 to 21%, and 77.27 to 78.49%, respectively. All these 11 bacteriophages belonged to the *Tempevirinae* subfamily within the *Drexlerviridae* family and showed similarity to the *T1svirus* genus (Supplementary Table S3). Phylogenomic comparison of bacteriophage HCF1 with these 11 bacteriophages as shown in the GBDP tree based on whole genomic data revealed that HCF1 formed its own distinct clade (Figure 5A). Phylogenetic analysis based on individual proteins (capsid and scaffold protein and portal protein) carried out using the GGDC web server considering ML and MP approaches also resulted in a distinct clade for the HCF1 (Figures 5B,C). The percentage similarity matrix generated using Clustal Omega with whole-genome sequence, capsid and scaffold protein, and portal protein sequences separately also showed that the HCF1 is distinct with a maximum percent similarity of 66, 58, and 67%, respectively, only (Figures 6A–C). To our knowledge, only three species of bacteriophages have been reported so far, namely, Phage Stevie from United States (Pasechnek et al., 2020), Phage CF1 DK-2017 from South Korea (Shaw et al., 2015), and Phage Sazh from United States (Kim et al., 2018) belonging to the *Tempevirinae* subfamily of the *Drexlerviridae* family which can infect *Citrobacter* spp. Among the 11 members of the *Drexlerviridae* family that were included for comparison, only one bacteriophage (*Salmonella* spp. phage 36) was from the Indian sub-continent (Karpe et al., 2016). However, this bacteriophage belonged to *Salmonella* host. Besides, there is one recently published report on bacteriophages infecting *C. amalonaticus* (Royam and Nachimuthu, 2020). The discrimination of species criterion at the nucleotide level for bacteriophage genome is set at 95% identity (Adriaenssens and Brister, 2017). Moreover, the comparative analysis of HCF1 with other bacteriophages with respect to Core-Genes suggested the presence of various common putative proteins among seven bacteriophages showing coverage up to 77.63, 51.72, 50, 54.22, 51.72, 42.86, 44.32, and 44.83% of protein profiles of bacteriophage CF1 DK-2017, virus Stevie, bacteriophage phSE-2, virus TLS, bacteriophage 36, bacteriophage LL5, and bacteriophage Sazh, respectively (Supplementary Table S4). It has been suggested that bacteriophages can be grouped together when they share ≥ 40% of core proteins with each other (Lavigne et al., 2008, 2009). According to this cut-off value, bacteriophage HCF1 may be grouped in the genus *T1svirus* along with these seven bacteriophages. This analysis also indicted that these eight bacteriophages including HCF1 shared moderate homology (77.63 to 42.86%) with each other at protein level. Taken together the data pertaining to nucleotide and protein profiles, we therefore propose that *Citrobacter* spp. virus HCF1 is a novel bacteriophage under the *Tempevirinae* subfamily of the *Drexlerviridae* family.

## Conclusion

In conclusion, we have isolated a novel virulent bacteriophage capable of killing the bacterium *C. amalonaticus* as well as *C. freundii.* The detailed analysis of its whole-genome properties and putative protein profiling as elucidated using various bioinformatic tools has allowed us to propose it as a novel species of bacteriophage belonging to the genus *T1svirus* under *Tempevirinae* subfamily within the *Drexlerviridae* family. The novel bacteriophage HCF1 exhibits no significant loss of viability up to 75 D when stored at 4°C. In addition, it tolerates high temperature (50°C) and alkaline pH as high as 11 without significant loss of the viability. Furthermore, *in vitro* efficacy assay indicated its potential for combating infections caused by *C. amalonaticus* and *C. freundii*. The predicted presence of DAM (*orf*55) in HCF1 suggests that it is able to evade the host R-M system. This study may further be extended for devising suitable strategies for the field applications of bacteriophage for validating its use as antimicrobial agent against *Citrobacter* spp.

## Data Availability Statement

The datasets presented in this study can be found in online repositories. The names of the repository/repositories and accession number(s) can be found in the article/Supplementary Material.

## Author Contributions

MM and DK conceived and designed the study. PK and MM performed the experiments, analyzed the data, and prepared the initial draft of the manuscript. All authors checked and reviewed the manuscript.

## Conflict of Interest

The authors declare that the research was conducted in the absence of any commercial or financial relationships that could be construed as a potential conflict of interest.

## Footnotes

1 http://rast.nmpdr.org/rast.cgi

2 https://blast.ncbi.nlm.nih.gov/Blast.cgi?PAGE=Protein

3 http://pfam.xfam.org/

4 https://www.ebi.ac.uk/interpro

5 http://biodev.cea.fr/virfam/

6 http://www.cbs.dtu.dk/services/TMHMM/

7 http://cgview.ca/

8 http://lowelab.ucsc.edu/tRNAscan-SE/

9 https://ggdc.dsmz.de/

10 https://www.ebi.ac.uk/Tools/msa/clustalo/
