# Supplementary material for: Phenotypic Characterization and Whole-Genome Analysis of a Novel Bacteriophage HCF1 Infecting Citrobacter amalonaticus and C. freundii
Source: Front Microbiol. 2021 Jan 25;12:644013. doi: 10.3389/fmicb.2021.644013 (PMC7868345; doi:10.3389/fmicb.2021.644013)
Supplement: Supplementary file 1 [file Data_Sheet_1.docx]

MS Title: Phenotypic characterization and whole genome analysis of a novel bacteriophage HCF1 infecting *Citrobacter amalonaticus* and *C. freundii*

Prince Kumar, Mukesh K. Meghvansi, D. V. Kamboj*

Biotechnology Division, Defence Research & Development Establishment, Gwalior 474002, Madhya Pradesh, India

*****Correspondingauthor

E-mail: [dvkamboj@gmail.com](mailto:dvkamboj@gmail.com)

**Supplementary Data (Figures S1 to S3; Tables S1 to S4)**


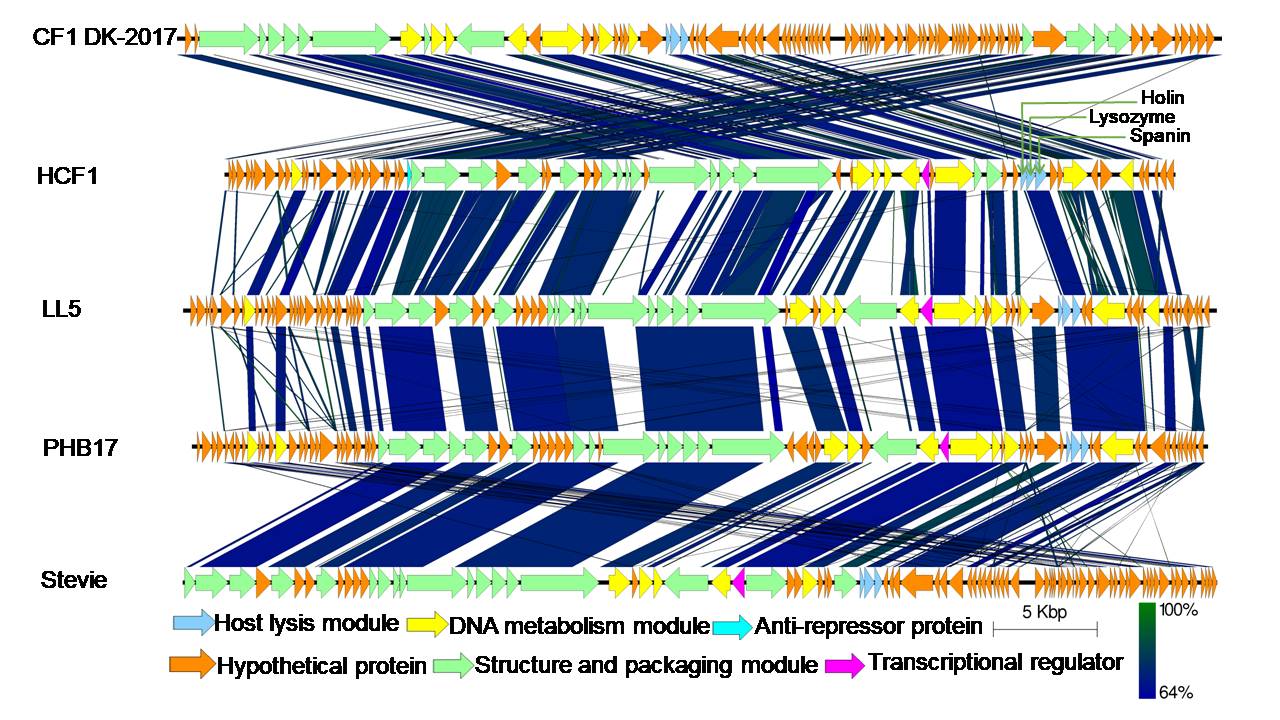


**SUPPLEMENTARY FIGURE S1 |** Comparative analysis of various modules of HCF1 with related four bacteriophages using EasyFig. Proposed functional module clusters are indicated by the same colour. Boxed arrows depict the position and transcriptional direction of ORFs and lines between genome maps indicate levels of homology.

**SUPPLEMENTARY FIGURE S2 |** Structure/function of *orf*59 of bacteriophage HCF1 as predicted using Phyre2 server. Putatively, *orf*59 indicates presence of lysozyme in HCF1 which could play important role in preventing premature lysis of the infected host cell.


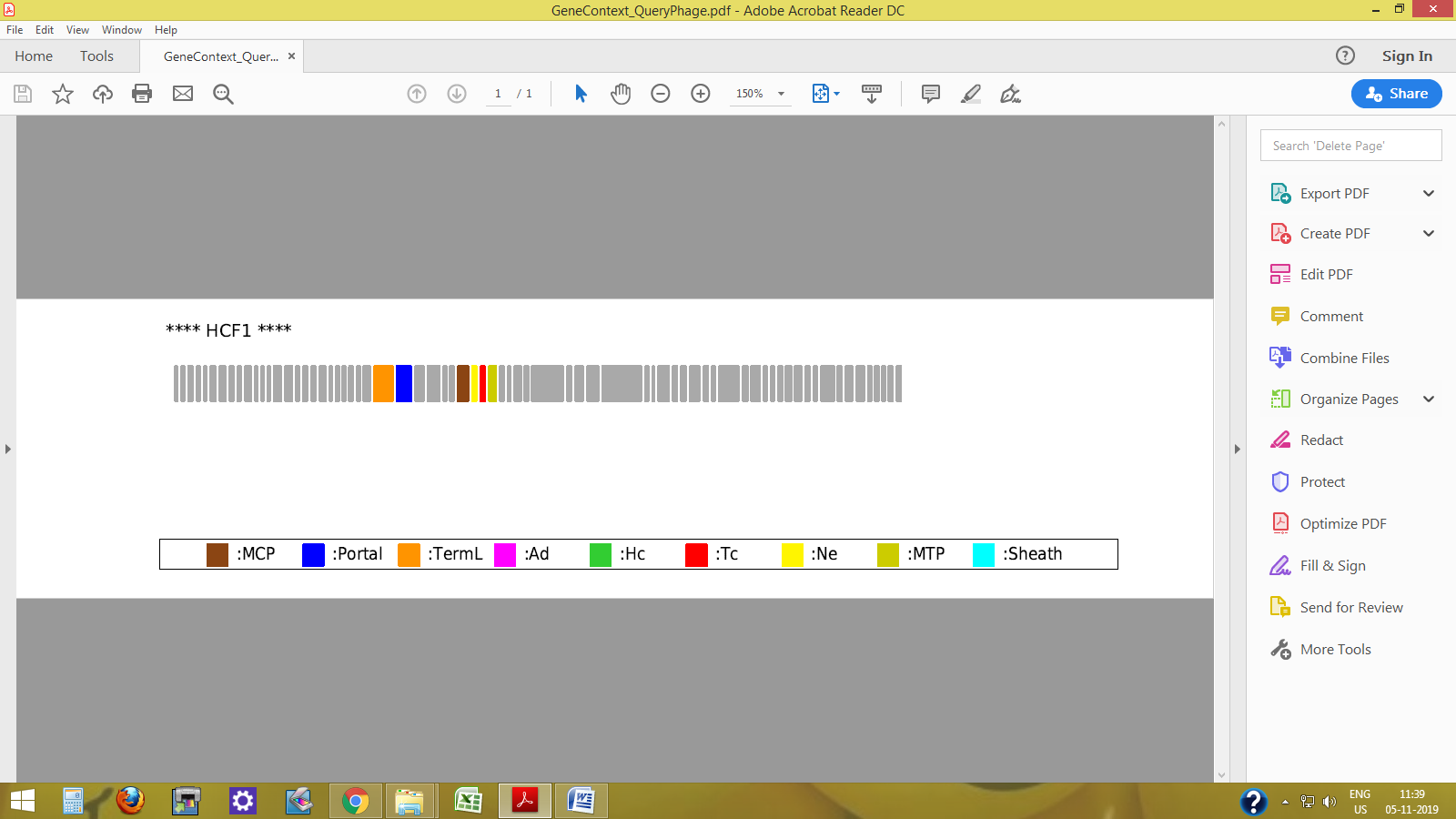


**SUPPLEMENTARY FIGURE S3 |** VirFam analysis of bacteriophage HCF1 head, neck and tail module.

**SUPPLEMENTARY TABLE** S**1 | General features of putative ORFs of bacteriophage HCF1, functional assignments, and homology to proteins in NCBI database.**

| **ORF** | **Locus tag** | **Protein ID** | **Coordinate** | **Strand** | **aa*** | **Predicted function, phage name and Closest protein match with accession number** | **E-value** | **%**  **Identity** |
| --- | --- | --- | --- | --- | --- | --- | --- | --- |
| ORF1 | HCF1_01 | [QGF21200](https://www.ncbi.nlm.nih.gov/protein/1774219561) | 205...375 | + | 56 | Hypothetical protein | 0 | 0 |
| ORF2 | HCF1_02 | [QGF21201](https://www.ncbi.nlm.nih.gov/protein/1774219561) | 365...583 | + | 72 | Hypothetical protein (*Escherichia* phage Henu7) ,QEA09680.1 | 6e-07 | 44.62 |
| ORF3 | HCF1_03 | [QGF21202](https://www.ncbi.nlm.nih.gov/protein/1774219561) | 599...982 | + | 127 | Hypothetical protein (Enterobacteria phage vB_EcoS_IME347) , AWD92208.1 | 0.072 | 41.67 |
| ORF4 | HCF1_04 | [QGF21203](https://www.ncbi.nlm.nih.gov/protein/1774219561) | 1048...1296 | + | 82 | Hypothetical protein | **-** | **-** |
| ORF5 | HCF1_05 | [QGF21204](https://www.ncbi.nlm.nih.gov/protein/1774219561) | 1283...1444 | + | 53 | Hypothetical protein | **-** | **-** |
| ORF6 | HCF1_06 | [QGF21205](https://www.ncbi.nlm.nih.gov/protein/1774219561) | 1444...1905 | + | 153 | Hypothetical protein phi2457T_0015 (*Shigella* phage phi2457T) , AYP69380.1 | 0.066 | 30.77 |
| ORF7 | HCF1_07 | [QGF21206](https://www.ncbi.nlm.nih.gov/protein/1774219561) | 1957...2484 | + | 175 | gp06 (*Escherichia* virus TLS) , YP_001285495.1 | 2E-21 | 38.19 |
| ORF8 | HCF1_08 | [QGF21207](https://www.ncbi.nlm.nih.gov/protein/1774219561) | 2598...2909 | + | 103 | Hypothetical protein JK16_00040 (*Shigella* phage JK16) , QEG04995.1 | 1E-11 | 46.34 |
| ORF9 | HCF1_09 | [QGF21208](https://www.ncbi.nlm.nih.gov/protein/1774219561) | 2930...3205 | + | 91 | Hypothetical protein vBEcoSW011D_28 (*Escherichia* phage vB_EcoS_W011D) , QCW18477.1 | 0.7 | 54.55 |
| ORF10 | HCF1_10 | [QGF21209](https://www.ncbi.nlm.nih.gov/protein/1774219561) | 3236...3766 | + | 176 | Polynucleotide kinase (*Escherichia* phage vB_EcoS_PHB17) , QDH94212.1 | 3E-91 | 74.01 |
| ORF11 | HCF1_11 | [QGF21210](https://www.ncbi.nlm.nih.gov/protein/1774219561) | 3763...3939 | + | 58 | Hypothetical protein CPT_LL5_10 (*Escherichia* phage LL5) , AWY04312.1 | 1E-24 | 77.59 |
| ORF12 | HCF1_12 | [QGF21211](https://www.ncbi.nlm.nih.gov/protein/1774219561) | 3945...4076 | + | 43 | Hypothetical protein | **-** | **-** |
| ORF13 | HCF1_13 | [QGF21212](https://www.ncbi.nlm.nih.gov/protein/1774219561) | 4304...4465 | + | 53 | gp13 (*Escherichia* virus TLS) , YP_001285502.1 | 3E-10 | 52.94 |
| ORF14 | HCF1_14 | [QGF21213](https://www.ncbi.nlm.nih.gov/protein/1774219561) | 4587...5246 | + | 219 | Hypothetical protein SP126_00225 (*Salmonella* virus SP126) , YP_009618021.1 | 1E-37 | 55.47 |
| ORF15 | HCF1_15 | [QGF21214](https://www.ncbi.nlm.nih.gov/protein/1774219561) | 5395...6012 | + | 205 | Hypothetical protein (*Citrobacter* phage CF1 DK-2017) , ARK07662.1 | 1E-16 | 42.34 |
| ORF16 | HCF1_16 | [QGF21215](https://www.ncbi.nlm.nih.gov/protein/1774219561) | 6080...6292 | + | 70 | Hypothetical protein CPT_Skenny_019 (*Klebsiella* phage Skenny) , QEG07202.1 | 4E-18 | 57.14 |
| ORF17 | HCF1_17 | [QGF21216](https://www.ncbi.nlm.nih.gov/protein/1774219561) | 6289...6660 | + | 123 | Hypothetical protein (*Salmonella* phage phSE-2) , YP_009280728.1 | 6E-17 | 81.82 |
| ORF18 | HCF1_18 | [QGF21217](https://www.ncbi.nlm.nih.gov/protein/1774219561) | 6677...7015 | + | 112 | Hypothetical protein CPT_Stevie83 (*Citrobacter* virus Stevie) , YP_009148789.1 | 0.001 | 64.29 |
| ORF19 | HCF1_19 | [QGF21218](https://www.ncbi.nlm.nih.gov/protein/1774219561) | 7012...7551 | + | 179 | Hypothetical protein (*Citrobacter* phage CF1 DK-2017) , ARK07665.1 | 1E-42 | 83.95 |
| ORF20 | HCF1_20 | [QGF21219](https://www.ncbi.nlm.nih.gov/protein/1774219561) | 7664...7807 | + | 47 | Hypothetical protein pSf1_0055 (*Shigella* phage pSf-1) , YP_008059777.1 | 2E-14 | 77.5 |
| ORF21 | HCF1_21 | [QGF21220](https://www.ncbi.nlm.nih.gov/protein/1774219561) | 7795...8022 | + | 75 | Hypothetical protein (*Salmonella* phage vB_SenS_PHB07) , AVQ09807.1 | 2E-39 | 78.67 |
| ORF22 | HCF1_22 | [QGF21221](https://www.ncbi.nlm.nih.gov/protein/1774219561) | 8206...8385 | + | 59 | Hypothetical protein CPT_LL5_27 (*Escherichia* phage LL5) , AWY04329.1 | 1E-23 | 82.69 |
| ORF23 | HCF1_23 | [QGF21222](https://www.ncbi.nlm.nih.gov/protein/1774219561) | 8387...8692 | + | 101 | Hypothetical protein YSP2_74 (*Salmonella* phage YSP2) , ATW57819.1 | 2E-17 | 50 |
| ORF24 | HCF1_24 | [QGF21223](https://www.ncbi.nlm.nih.gov/protein/1774219561) | 8815...9012 | + | 65 | Antirepressor (*Salmonella* phage 36) , YP_009223431.1 | 0.02 | 68 |
| ORF25 | HCF1_25 | [QGF21224](https://www.ncbi.nlm.nih.gov/protein/1774219561) | 9005...9556 | + | 183 | Terminase small subunit (*Salmonella* phage phSE-2) , YP_009280735.1 | 5E-26 | 78.79 |
| ORF26 | HCF1_26 | [QGF21225](https://www.ncbi.nlm.nih.gov/protein/1774219561) | 9629...11386 | + | 585 | Putative terminase large subunit (*Salmonella* virus SP126) , YP_009618006.1 | 1E-99 | 90.18 |
| ORF27 | HCF1_27 | [QGF21226](https://www.ncbi.nlm.nih.gov/protein/1774219561) | 11761...13122 | + | 453 | Putative portal protein (*Citrobacter* phage Sazh) , AXY85458.1 | 7E-140 | 58.2 |
| ORF28 | HCF1_28 | [QGF21227](https://www.ncbi.nlm.nih.gov/protein/1774219561) | 13112...13852 | + | 246 | Putative phage head morphogenesis protein (*Salmonella* virus SP126) , YP_009618004.1 | 2E-74 | 67.78 |
| ORF29 | HCF1_29 | [QGF21228](https://www.ncbi.nlm.nih.gov/protein/1774219561) | 14173...15276 | + | 367 | Capsid and scaffold protein (*Salmonella* phage phSE-2) , YP_009280739.1 | 3E-117 | 64.57 |
| ORF30 | HCF1_30 | [QGF21229](https://www.ncbi.nlm.nih.gov/protein/1774219561) | 15309...15500 | + | 63 | Hypothetical protein | **-** | **-** |
| ORF31 | HCF1_31 | [QGF21230](https://www.ncbi.nlm.nih.gov/protein/1774219561) | 15502...15780 | + | 92 | Hypothetical protein | **-** | **-** |
| ORF32 | HCF1_32 | [QGF21231](https://www.ncbi.nlm.nih.gov/protein/1774219561) | 16158...17111 | + | 317 | Major capsid protein (*Salmonella* virus SP126) , YP_009618000.1 | 3E-64 | 50 |
| ORF33 | HCF1_33 | [QGF21232](https://www.ncbi.nlm.nih.gov/protein/1774219561) | 17320...17682 | + | 120 | Hypothetical protein GJL01_16 (*Salmonella* phage GJL01) , ARB06641.1 | 1E-73 | 89.17 |
| ORF34 | HCF1_34 | [QGF21233](https://www.ncbi.nlm.nih.gov/protein/1774219561) | 17782...18159 | + | 125 | gp41 (*Escherichia* virus TLS) , YP_001285530.1 | 1E-44 | 74.47 |
| ORF35 | HCF1_35 | [QGF21234](https://www.ncbi.nlm.nih.gov/protein/1774219561) | 18170...18793 | + | 207 | Phage tail tube protein (*Salmonella* phage 36) ,, YP_009223452.1 | 9E-45 | 61.73 |
| ORF36 | HCF1_36 | [QGF21235](https://www.ncbi.nlm.nih.gov/protein/1774219561) | 18900...19205 | + | 101 | Putative tape measure chaperone protein (*Escherichia* phage Henu7) , QEA09710.1 | 8E-26 | 54.64 |
| ORF37 | HCF1_37 | [QGF21236](https://www.ncbi.nlm.nih.gov/protein/1774219561) | 19332...19529 | + | 65 | TfmB (*Escherichia* virus TLS) , YP_001285534.1 | 5E-37 | 90.77 |
| ORF38 | HCF1_38 | [QGF21237](https://www.ncbi.nlm.nih.gov/protein/1774219561) | 19562...20119 | + | 185 | Putative minor tail fiber protein (*Salmonella* phage 36) , YP_009223456.1 | 3E-12 | 78.05 |
| ORF39 | HCF1_39 | [QGF21238](https://www.ncbi.nlm.nih.gov/protein/1774219561) | 20216...20476 | + | 86 | Hypothetical protein | **-** | **-** |
| ORF40 | HCF1_40 | [QGF21239](https://www.ncbi.nlm.nih.gov/protein/1774219561) | 20473...23409 | + | 978 | Tail length tape-measure protein (*Escherichia* phage Jahat_MG145) , QBZ71364.1 | 0 | 42.73 |
| ORF41 | HCF1_41 | [QGF21240](https://www.ncbi.nlm.nih.gov/protein/1774219561) | 23402...23767 | + | 121 | Phage tail fiber protein, gp46 (Escherichia virus TLS) , YP_001285536.1 | 2E-45 | 60.18 |
| ORF42 | HCF1_42 | [QGF21241](https://www.ncbi.nlm.nih.gov/protein/1774219561) | 23843...24541 | + | 232 | Putative minor tail protein (*Escherichia* phage vB_EcoS_G29-2) , QBQ81497.1 | 8E-44 | 60.94 |
| ORF43 | HCF1_43 | [QGF21242](https://www.ncbi.nlm.nih.gov/protein/1774219561) | 24543...25559 | + | 338 | Tail assembly protein (Escherichia phage LL5) , AWY04352.1 | 6E-87 | 68.34 |
| ORF44 | HCF1_44 | [QGF21243](https://www.ncbi.nlm.nih.gov/protein/1774219561) | 25631...29347 | + | 1238 | Putative phage tail protein, TspJ (*Escherichia* virus TLS) , YP_001285540.1 | 0 | 67.48 |
| ORF45 | HCF1_45 | [QGF21244](https://www.ncbi.nlm.nih.gov/protein/1774219561) | 29420...29671 | - | 83 | Hypothetical protein pSf2_020 (*Shigella* phage pSf-2) , YP_009112958.1 | 0.001 | 46.51 |
| ORF46 | HCF1_46 | [QGF21245](https://www.ncbi.nlm.nih.gov/protein/1774219561) | 30121...30234 | - | 37 | Hypothetical protein | **-** | **-** |
| ORF47 | HCF1_47 | [QGF21246](https://www.ncbi.nlm.nih.gov/protein/1774219561) | 30246...31232 | + | 328 | Putative exodeoxyribonuclease VIII (*Salmonella* phage YSP2) , ATW57796.1 | 3E-145 | 61.67 |
| ORF48 | HCF1_48 | [QGF21247](https://www.ncbi.nlm.nih.gov/protein/1774219561) | 31261...31605 | + | 114 | Putative recombination protein (*Salmonella* phage 36) , YP_009223471.1 | 1E-18 | 82.22 |
| ORF49 | HCF1_49 | [QGF21248](https://www.ncbi.nlm.nih.gov/protein/1774219561) | 31789...32169 | + | 126 | Ssb (*Escherichia* virus TLS) , YP_001285544.1 | 1E-56 | 81.13 |
| ORF50 | HCF1_50 | [QGF21249](https://www.ncbi.nlm.nih.gov/protein/1774219561) | 32566...33465 | - | 299 | DNA primase/helicase (*Citrobacter* phage CF1 DK-2017) , ARK07610.1 | 3E-42 | 94.29 |
| ORF51 | HCF1_51 | [QGF21250](https://www.ncbi.nlm.nih.gov/protein/1774219561) | 33567...33947 | - | 126 | Putative transcriptional regulator (*Escherichia* phage vB_EcoS_W011D) , QCW18508.1 | 2E-25 | 46.72 |
| ORF52 | HCF1_52 | [QGF21251](https://www.ncbi.nlm.nih.gov/protein/1774219561) | 33999...34205 | + | 68 | Hypothetical protein | **-** | **-** |
| ORF53 | HCF1_53 | [QGF21252](https://www.ncbi.nlm.nih.gov/protein/1774219561) | 34247...36106 | + | 619 | ATP- dependent Helicase (*Citrobacter* virus Stevie) , YP_009148736.1 | 0 | 59.97 |
| ORF54 | HCF1_54 | [QGF21253](https://www.ncbi.nlm.nih.gov/protein/1774219561) | 36108...36503 | + | 131 | Putative endonuclease (*Salmonella* phage GJL01) , ARB06702.1 | 9E-29 | 57.3 |
| ORF55 | HCF1_55 | [QGF21254](https://www.ncbi.nlm.nih.gov/protein/1774219561) | 36714...37490 | + | 258 | DNA adenine methyltransferase (*Salmonella* phage vB_SenS_PHB07) , AVQ09766.1 | 7E-104 | 64.23 |
| ORF56 | HCF1_56 | [QGF21255](https://www.ncbi.nlm.nih.gov/protein/1774219561) | 37490...37711 | + | 73 | Hypothetical protein SP126_00380 (*Salmonella* virus SP126) , YP_009618052.1 | 5E-13 | 43.48 |
| ORF57 | HCF1_57 | [QGF21256](https://www.ncbi.nlm.nih.gov/protein/1774219561) | 37989...38252 | + | 87 | Hypothetical protein (*Salmonella* phage vB_SenS_PHB07) , AVQ09763.1 | 4E-18 | 62.5 |
| ORF58 | HCF1_58 | [QGF21257](https://www.ncbi.nlm.nih.gov/protein/1774219561) | 38363...38662 | + | 99 | Holin (*Salmonella* phage vB_SenS_PHB07) , AVQ09761.1 | 4E-16 | 53.52 |
| ORF59 | HCF1_59 | [QGF21258](https://www.ncbi.nlm.nih.gov/protein/1774219561) | 38634...39071 | + | 145 | Lysozyme (*Escherichia* phage vB_EcoS_PHB17) , QDH94273.1 | 3E-55 | 54.79 |
| ORF60 | HCF1_60 | [QGF21259](https://www.ncbi.nlm.nih.gov/protein/1774219561) | 39068...39613 | + | 181 | Unimolecular spanin protein (*Escherichia* phage LL5) , AWY04374.1 | 3E-61 | 90.38 |
| ORF61 | HCF1_61 | [QGF21260](https://www.ncbi.nlm.nih.gov/protein/1774219561) | 39773...40150 | + | 125 | Hypothetical protein YSP2_20 (*Salmonella* phage YSP2) , ATW57776.1 | 5E-17 | 61.67 |
| ORF62 | HCF1_62 | [QGF21261](https://www.ncbi.nlm.nih.gov/protein/1774219561) | 40147...40392 | + | 81 | Hypothetical protein | **-** | **-** |
| ORF63 | HCF1_63 | [QGF21262](https://www.ncbi.nlm.nih.gov/protein/1774219561) | 40413...41600 | + | 395 | Helicase (*Salmonella* phage 36) , YP_009223509.1 | 2E-51 | 73.45 |
| ORF64 | HCF1_64 | [QGF21263](https://www.ncbi.nlm.nih.gov/protein/1774219561) | 41737...42057 | - | 106 | Hypothetical protein GJL01_66 (*Salmonella* phage GJL01) , ARB06691.1 | 2E-25 | 60 |
| ORF65 | HCF1_65 | [QGF21264](https://www.ncbi.nlm.nih.gov/protein/1774219561) | 42202...42786 | + | 194 | Hypothetical protein (*Salmonella* phage vB_SenS_PHB07) , AVQ09754.1 | 1E-36 | 50.31 |
| ORF66 | HCF1_66 | [QGF21265](https://www.ncbi.nlm.nih.gov/protein/1774219561) | 43087...43767 | - | 226 | Site-specific DNA-cytosine methylase (*Salmonella* phage vB_SenS_PHB07) , AVQ09753.1 | 2E-102 | 79.77 |
| ORF67 | HCF1_67 | [QGF21266](https://www.ncbi.nlm.nih.gov/protein/1774219561) | 44028...44267 | - | 79 | Hypothetical protein CPT_LL5_80 (*Escherichia* phage LL5) , AWY04382.1 | 3E-29 | 63.29 |
| ORF68 | HCF1_68 | [QGF21267](https://www.ncbi.nlm.nih.gov/protein/1774219561) | 44264...44521 | - | 85 | Hypothetical protein | **-** | **-** |
| ORF69 | HCF1_69 | [QGF21268](https://www.ncbi.nlm.nih.gov/protein/1774219561) | 44792...44962 | - | 56 | Hypothetical protein NBD2_80 (Enterobacteria phage vB_EcoS_NBD2) , YP_009284704.1 | 2E-14 | 83.78 |
| ORF70 | HCF1_70 | [QGF21269](https://www.ncbi.nlm.nih.gov/protein/1774219561) | 45043...45366 | - | 107 | Hypothetical protein (*Escherichia* phage SRT8) , YP_009615464.1 | 2E-20 | 67.74 |
| ORF71 | HCF1_71 | [QGF21270](https://www.ncbi.nlm.nih.gov/protein/1774219561) | 45363...45725 | - | 120 | Hypothetical protein YSP2_01 (*Salmonella* phage YSP2) , ATW57760.1 | 2e-09 | 63.04 |

*Amino acid

**SUPPLEMENTARY TABLE** S**2|** Highest identities of proteins of HCF1 with at least three proteins taken from ACLAME database of bacteriophages as revealed through VirFam analysis.

| **Protein**  **Super family** | **Corresponding**  **Protein in ACLAME** | **ACLAME Phage** | **Seq. Identity (%)** |
| --- | --- | --- | --- |
| [MCP](http://biodev.cea.fr/virfam/files/8cf5bc68-f041-4776-bb6c-050c178ca546/help/Morphogenesis_assembly.html) | [protein:vir:79642](http://aclame.ulb.ac.be/perl/Aclame/Genomes/prot_view.cgi?view=prot&id=protein:vir:79642) [protein:vir:107687](http://aclame.ulb.ac.be/perl/Aclame/Genomes/prot_view.cgi?view=prot&id=protein:vir:107687) [protein:vir:103285](http://aclame.ulb.ac.be/perl/Aclame/Genomes/prot_view.cgi?view=prot&id=protein:vir:103285) | [TLS](http://biodev.cea.fr/virfam/files/8cf5bc68-f041-4776-bb6c-050c178ca546/NC_009540_mge_1872/results_CNT.html) [T1](http://biodev.cea.fr/virfam/files/8cf5bc68-f041-4776-bb6c-050c178ca546/NC_005833_mge_1518/results_CNT.html) [JK06](http://biodev.cea.fr/virfam/files/8cf5bc68-f041-4776-bb6c-050c178ca546/NC_007291_mge_1605/results_CNT.html) | 68 66 50 |
| [Portal](http://biodev.cea.fr/virfam/files/8cf5bc68-f041-4776-bb6c-050c178ca546/help/Morphogenesis_assembly.html) | [protein:vir:103219](http://aclame.ulb.ac.be/perl/Aclame/Genomes/prot_view.cgi?view=prot&id=protein:vir:103219) [protein:vir:79647](http://aclame.ulb.ac.be/perl/Aclame/Genomes/prot_view.cgi?view=prot&id=protein:vir:79647) [protein:vir:107662](http://aclame.ulb.ac.be/perl/Aclame/Genomes/prot_view.cgi?view=prot&id=protein:vir:107662) | [JK06](http://biodev.cea.fr/virfam/files/8cf5bc68-f041-4776-bb6c-050c178ca546/NC_007291_mge_1605/results_CNT.html) [TLS](http://biodev.cea.fr/virfam/files/8cf5bc68-f041-4776-bb6c-050c178ca546/NC_009540_mge_1872/results_CNT.html) [T1](http://biodev.cea.fr/virfam/files/8cf5bc68-f041-4776-bb6c-050c178ca546/NC_005833_mge_1518/results_CNT.html) | 59 53 52 |
| [TermL](http://biodev.cea.fr/virfam/files/8cf5bc68-f041-4776-bb6c-050c178ca546/help/Morphogenesis_assembly.html) | [protein:vir:103223](http://aclame.ulb.ac.be/perl/Aclame/Genomes/prot_view.cgi?view=prot&id=protein:vir:103223) [protein:vir:79648](http://aclame.ulb.ac.be/perl/Aclame/Genomes/prot_view.cgi?view=prot&id=protein:vir:79648) [protein:vir:107694](http://aclame.ulb.ac.be/perl/Aclame/Genomes/prot_view.cgi?view=prot&id=protein:vir:107694) | [JK06](http://biodev.cea.fr/virfam/files/8cf5bc68-f041-4776-bb6c-050c178ca546/NC_007291_mge_1605/results_CNT.html) [TLS](http://biodev.cea.fr/virfam/files/8cf5bc68-f041-4776-bb6c-050c178ca546/NC_009540_mge_1872/results_CNT.html) [T1](http://biodev.cea.fr/virfam/files/8cf5bc68-f041-4776-bb6c-050c178ca546/NC_005833_mge_1518/results_CNT.html) | 42 36 31 |
| [MTP](http://biodev.cea.fr/virfam/files/8cf5bc68-f041-4776-bb6c-050c178ca546/help/Morphogenesis_assembly.html) | [protein:vir:79636](http://aclame.ulb.ac.be/perl/Aclame/Genomes/prot_view.cgi?view=prot&id=protein:vir:79636) [protein:vir:107670](http://aclame.ulb.ac.be/perl/Aclame/Genomes/prot_view.cgi?view=prot&id=protein:vir:107670) [protein:vir:103277](http://aclame.ulb.ac.be/perl/Aclame/Genomes/prot_view.cgi?view=prot&id=protein:vir:103277) | [TLS](http://biodev.cea.fr/virfam/files/8cf5bc68-f041-4776-bb6c-050c178ca546/NC_009540_mge_1872/results_CNT.html) [T1](http://biodev.cea.fr/virfam/files/8cf5bc68-f041-4776-bb6c-050c178ca546/NC_005833_mge_1518/results_CNT.html) [JK06](http://biodev.cea.fr/virfam/files/8cf5bc68-f041-4776-bb6c-050c178ca546/NC_007291_mge_1605/results_CNT.html) | 50 47 39 |
| [Ne1](http://biodev.cea.fr/virfam/files/8cf5bc68-f041-4776-bb6c-050c178ca546/help/Morphogenesis_assembly.html) | [protein:vir:79638](http://aclame.ulb.ac.be/perl/Aclame/Genomes/prot_view.cgi?view=prot&id=protein:vir:79638) [protein:vir:107703](http://aclame.ulb.ac.be/perl/Aclame/Genomes/prot_view.cgi?view=prot&id=protein:vir:107703) [protein:vir:103280](http://aclame.ulb.ac.be/perl/Aclame/Genomes/prot_view.cgi?view=prot&id=protein:vir:103280) | [TLS](http://biodev.cea.fr/virfam/files/8cf5bc68-f041-4776-bb6c-050c178ca546/NC_009540_mge_1872/results_CNT.html) [T1](http://biodev.cea.fr/virfam/files/8cf5bc68-f041-4776-bb6c-050c178ca546/NC_005833_mge_1518/results_CNT.html) [JK06](http://biodev.cea.fr/virfam/files/8cf5bc68-f041-4776-bb6c-050c178ca546/NC_007291_mge_1605/results_CNT.html) | 88 62 44 |
| [Tc1](http://biodev.cea.fr/virfam/files/8cf5bc68-f041-4776-bb6c-050c178ca546/help/Morphogenesis_assembly.html) | [protein:vir:79637](http://aclame.ulb.ac.be/perl/Aclame/Genomes/prot_view.cgi?view=prot&id=protein:vir:79637) [protein:vir:107704](http://aclame.ulb.ac.be/perl/Aclame/Genomes/prot_view.cgi?view=prot&id=protein:vir:107704) [protein:vir:104348](http://aclame.ulb.ac.be/perl/Aclame/Genomes/prot_view.cgi?view=prot&id=protein:vir:104348) | [TLS](http://biodev.cea.fr/virfam/files/8cf5bc68-f041-4776-bb6c-050c178ca546/NC_009540_mge_1872/results_CNT.html) [T1](http://biodev.cea.fr/virfam/files/8cf5bc68-f041-4776-bb6c-050c178ca546/NC_005833_mge_1518/results_CNT.html) [RTP](http://biodev.cea.fr/virfam/files/8cf5bc68-f041-4776-bb6c-050c178ca546/NC_007603_mge_1593/results_CNT.html) | 61 50 41 |

**SUPPLEMENTARY TABLE** S**3|** Comparison of taxonomic profile and genome features of bacteriophage HCF1 with related bacteriophages available in NCBI database showing E-value 0.0.

| S No. | Name of phage | Genus | Subfamily | Family | Query coverage (%) | E value | % Identity | GC  Content  (%) | Size  (kbp) | ORF |
| --- | --- | --- | --- | --- | --- | --- | --- | --- | --- | --- |
| 1 | *Citrobacter* phage CF1 DK-2017 | *T1svirus* | *Tempevirinae* | *Drexlerviridae* | 21 | 0.0 | 78.49 | 42.6 | 50.34 | 87 |
| 2 | *Salmonella* phage vB_SenS_PHB07 | *T1svirus* | *Tempevirinae* | *Drexlerviridae* | 20 | 0.0 | 77.78 | 43.4 | 51.82 | 87 |
| 3 | *Salmonella* phage phSE-5 | *T1svirus* | *Tempevirinae* | *Drexlerviridae* | 17 | 0.0 | 78.39 | 42.9 | 49.18 | 83 |
| 4 | *Salmonella* phage phSE-2 | *T1svirus* | *Tempevirinae* | *Drexlerviridae* | 17 | 0.0 | 78.39 | 42.9 | 49.17 | 83 |
| 5 | *Salmonella* phage YSP2 | *T1svirus* | *Tempevirinae* | *Drexlerviridae* | 18 | 0.0 | 78.38 | 42.9 | 50.32 | 87 |
| 6 | *Salmonella* phage GJL01 | *T1svirus* | *Tempevirinae* | *Drexlerviridae* | 18 | 0.0 | 78.32 | 42.8 | 50.41 | 80 |
| 7 | *Citrobacter* virus Stevie | *T1svirus* | *Tempevirinae* | *Drexlerviridae* | 20 | 0.0 | 78.20 | 42.8 | 49.82 | 90 |
| 8 | *Escherichia* virus TLS | *T1svirus* | *Tempevirinae* | *Drexlerviridae* | 19 | 0.0 | 78.21 | 42.7 | 49.9 | 87 |
| 9 | *Salmonella* phage 36 | *T1svirus* | *Tempevirinae* | *Drexlerviridae* | 15 | 0.0 | 77.27 | 43.5 | 41.09 | 91 |
| 10 | *Citrobacter* phage Sazh | *T1svirus* | *Tempevirinae* | *Drexlerviridae* | 20 | 0.0 | 78.04 | 42.8 | 49.67 | 87 |
| 11 | *Escherichia* phage LL5 | *T1svirus* | *Tempevirinae* | *Drexlerviridae* | 18 | 0.0 | 77.96 | 42.5 | 49.79 | 88 |
| 12 | *Citrobacter virus HFC1* | *T1svirus* | *Tempevirinae* | *Drexlerviridae* | Described in this study | | | 44.5 | 45.8 | 71 |

Note: Data retrieved from NCBI database on 08 June, 2020.

**SUPPLEMENTARY TABLE** S**4|** Core-Gene analysis of bacteriophage HCF1 with other similar Phage of genus *T1svirus*.

| **S. No.** | **Virus HCF1**  **(MN545971)** | **Phage CF1 DK-2017 (NC_047823)** | **Virus Stevie (NC_027350)** | **Phage phSE-2 (KX015770)** | **Virus TLS (AY308796)** | **Phage 36 (KR296690)** | **Phage LL5 (NC_047985)** | **Phage Sazh (NC_048065)** |  |
| --- | --- | --- | --- | --- | --- | --- | --- | --- | --- |
|  | NCBI Protein ID | NCBI Protein ID | NCBI Protein ID | NCBI Protein ID | NCBI Protein ID | NCBI Protein ID | NCBI Protein ID | NCBI Protein ID |  |
| 1 | - | - | - | - | - | AKJ73973.1 | YP_009802523.1 | YP_009812443.1 |  |
| 2 | QGF21201.1 | YP_009790256.1 | YP_009148778.1 | ANA49760.1 | AAR09240.1 | AKJ73974.1 | YP_009802524.1 | YP_009812444.1 |  |
| 3 | QGF21209.1 | YP_009790255.1 | YP_009148777.1 | ANA49759.1 | AAR09239.1 | AKJ73975.1 | YP_009802525.1 | YP_009812445.1 |  |
| 4 | QGF21213.1 | YP_009790260.1 | YP_009148782.1 | ANA49680.1 | AAR09244.1 | AKJ73976.1 | YP_009802526.1 | YP_009812446.1 |  |
| 5 | QGF21214.1 | YP_009790265.1 | YP_009148787.1 | ANA49685.1 | AAR09249.1 | AKJ73978.1 | YP_009802529.1 | YP_009812448.1 |  |
| 6 | QGF21218.1 | YP_009790268.1 | YP_009148790.1 | ANA49688.1 | AAR09252.1 | AKJ73979.1 | YP_009802530.1 | YP_009812449.1 |  |
| 7 | QGF21219.1 | YP_009790269.1 | YP_009148791.1 | ANA49689.1 | AAR09253.1 | AKJ73982.1 | YP_009802531.1 | YP_009812450.1 |  |
| 8 | QGF21220.1 | YP_009790270.1 | YP_009148792.1 | ANA49690.1 | AAR09254.1 | AKJ73984.1 | YP_009802532.1 | YP_009812451.1 |  |
| 9 | QGF21221.1 | YP_009790272.1 | YP_009148795.1 | ANA49692.1 | AAR09257.1 | AKJ73987.1 | YP_009802533.1 | YP_009812452.1 |  |
| 10 | QGF21222.1 | YP_009790273.1 | YP_009148796.1 | ANA49693.1 | AAR09258.1 | AKJ73989.1 | YP_009802534.1 | YP_009812453.1 |  |
| 11 | QGF21224.1 | YP_009790274.1 | YP_009148707.1 | ANA49694.1 | AAR09259.1 | AKJ73991.1 | YP_009802535.1 | YP_009812454.1 |  |
| 12 | QGF21225.1 | YP_009790275.1 | YP_009148708.1 | ANA49695.1 | AAR09260.1 | AKJ73996.1 | YP_009802538.1 | YP_009812457.1 |  |
| 13 | QGF21226.1 | YP_009790276.1 | YP_009148709.1 | ANA49696.1 | AAR09261.1 | AKJ74000.1 | YP_009802542.1 | YP_009812461.1 |  |
| 14 | QGF21227.1 | YP_009790277.1 | YP_009148710.1 | ANA49697.1 | AAR09262.1 | AKJ74001.1 | YP_009802543.1 | YP_009812462.1 |  |
| 15 | QGF21228.1 | YP_009790278.1 | YP_009148711.1 | ANA49698.1 | AAR09263.1 | - | - | - |  |
| 16 | QGF21231.1 | YP_009790281.1 | YP_009148714.1 | ANA49701.1 | AAR09266.1 | AKJ74002.1 | YP_009802545.1 | YP_009812463.1 |  |
| 17 | QGF21232.1 | YP_009790285.1 | YP_009148718.1 | ANA49705.1 | AAR09270.1 | AKJ74003.1 | YP_009802547.1 | YP_009812465.1 |  |
| 18 | QGF21233.1 | YP_009790286.1 | YP_009148719.1 | ANA49706.1 | AAR09271.1 | AKJ74005.1 | YP_009802546.1 | YP_009812464.1 |  |
| 19 | QGF21235.1 | YP_009790200.1 | YP_009148722.1 | ANA49708.1 | AAR09273.1 | AKJ74007.1 | YP_009802548.1 | YP_009812466.1 |  |
| 20 | QGF21236.1 | YP_009790201.1 | YP_009148721.1 | ANA49709.1 | AAR09274.1 | AKJ74011.1 | YP_009802549.1 | YP_009812467.1 |  |
| 21 | QGF21239.1 | YP_009790202.1 | YP_009148723.1 | ANA49710.1 | AAR09276.1 | AKJ74012.1 | YP_009802550.1 | YP_009812468.1 |  |
| 22 | QGF21240.1 | YP_009790203.1 | YP_009148724.1 | ANA49711.1 | AAR09277.1 | AKJ74013.1 | YP_009802551.1 | YP_009812469.1 |  |
| 23 | QGF21241.1 | YP_009790204.1 | YP_009148725.1 | ANA49712.1 | AAR09278.1 | AKJ74015.1 | YP_009802553.1 | YP_009812471.1 |  |
| 24 | QGF21242.1 | YP_009790205.1 | YP_009148726.1 | ANA49713.1 | AAR09279.1 | AKJ74019.1 | YP_009802555.1 | YP_009812472.1 |  |
| 25 | QGF21243.1 | YP_009790207.1 | YP_009148728.1 | ANA49715.1 | AAR09281.1 | AKJ74021.1 | YP_009802557.1 | YP_009812474.1 |  |
| 26 | QGF21246.1 | YP_009790208.1 | YP_009148729.1 | ANA49716.1 | AAR09282.1 | AKJ74023.1 | YP_009802558.1 | YP_009812475.1 |  |
| 27 | QGF21247.1 | YP_009790210.1 | YP_009148731.1 | ANA49718.1 | AAR09284.1 | AKJ74035.1 | YP_009802560.1 | YP_009812477.1 |  |
| 28 | QGF21248.1 | YP_009790211.1 | YP_009148732.1 | ANA49719.1 | AAR09285.1 | AKJ74038.1 | YP_009802561.1 | YP_009812478.1 |  |
| 29 | QGF21249.1 | YP_009790213.1 | YP_009148734.1 | ANA49721.1 | AAR09287.1 | AKJ74039.1 | YP_009802562.1 | YP_009812479.1 |  |
| 30 | QGF21250.1 | YP_009790214.1 | YP_009148735.1 | ANA49722.1 | AAR09288.1 | AKJ74041.1 | YP_009802563.1 | YP_009812480.1 |  |
| 31 | QGF21252.1 | YP_009790215.1 | YP_009148736.1 | ANA49723.1 | AAR09289.1 | AKJ74044.1 | YP_009802566.1 | YP_009812482.1 |  |
| 32 | QGF21253.1 | YP_009790216.1 | YP_009148737.1 | ANA49724.1 | AAR09290.1 | AKJ74046.1 | YP_009802567.1 | YP_009812483.1 |  |
| 33 | QGF21254.1 | YP_009790218.1 | YP_009148739.1 | ANA49726.1 | AAR09292.1 | AKJ74048.1 | YP_009802569.1 | YP_009812485.1 |  |
| 34 | QGF21255.1 | YP_009790219.1 | YP_009148740.1 | ANA49727.1 | AAR09293.1 | AKJ74052.1 | YP_009802572.1 | YP_009812487.1 |  |
| 35 | QGF21256.1 | YP_009790221.1 | YP_009148742.1 | ANA49729.1 | AAR09295.1 | AKJ74053.1 | YP_009802573.1 | YP_009812488.1 |  |
| 36 | QGF21257.1 | YP_009790224.1 | YP_009148744.1 | ANA49731.1 | AAR09298.1 | AKJ74055.1 | YP_009802574.1 | YP_009812489.1 |  |
| 37 | QGF21258.1 | YP_009790225.1 | YP_009148745.1 | ANA49732.1 | AAR09299.1 | AKJ74057.1 | YP_009802576.1 | YP_009812492.1 |  |
| 38 | QGF21259.1 | YP_009790226.1 | YP_009148746.1 | ANA49733.1 | AAR09300.1 | AKJ74059.1 | YP_009802577.1 | YP_009812493.1 |  |
| 39 | QGF21260.1 | YP_009790229.1 | YP_009148749.1 | ANA49736.1 | AAR09301.1 | AKJ74062.1 | YP_009802578.1 | YP_009812494.1 |  |
| 40 | QGF21262.1 | YP_009790230.1 | YP_009148750.1 | ANA49737.1 | AAR09302.1 | AKJ74063.1 | YP_009802579.1 | YP_009812495.1 |  |
| 41 | QGF21263.1 | YP_009790231.1 | YP_009148751.1 | ANA49738.1 | AAR09303.1 | - | - | - |  |
| 42 | QGF21264.1 | YP_009790233.1 | YP_009148752.1 | ANA49739.1 | AAR09304.1 | - | - | - |  |
| 43 | QGF21265.1 | YP_009790234.1 | YP_009148753.1 | ANA49740.1 | AAR09305.1 | - | - | - |  |
| 44 | QGF21266.1 | YP_009790235.1 | YP_009148754.1 | ANA49741.1 | AAR09306.1 | - | - | - |  |
| 45 | QGF21268.1 | YP_009790238.1 | YP_009148757.1 | ANA49744.1 | AAR09309.1 | - | - | - |  |
| 46 | QGF21269.1 | YP_009790241.1 | YP_009148760.1 | ANA49746.1 | AAR09311.1 | - | - | - |  |
